# SmCSN5 is a synergist in the transcription factor SmMYB36-mediated biosynthesis of tanshinones and phenolic acids in *Salvia miltiorrhiza*

**DOI:** 10.1093/hr/uhaf005

**Published:** 2025-01-06

**Authors:** Qi Li, Xiujuan Wang, Jie Wang, Yan Su, Yuanyi Guo, Jie Yang, Jingying Liu, Zheyong Xue, Juane Dong, Pengda Ma

**Affiliations:** College of Life Sciences, Northwest A&F University, No.22 Xinong Road, Yangling 712100, China; College of Tobacco Science, Yunnan Agricultural University, No. 452 Fengyuan Road, Panlong District, Kunming 650201, China; College of Life Sciences, Northwest A&F University, No.22 Xinong Road, Yangling 712100, China; College of Life Sciences, Northwest A&F University, No.22 Xinong Road, Yangling 712100, China; College of Life Sciences, Northwest A&F University, No.22 Xinong Road, Yangling 712100, China; College of Life Sciences, Northwest A&F University, No.22 Xinong Road, Yangling 712100, China; College of Life Sciences, Northwest A&F University, No.22 Xinong Road, Yangling 712100, China; College of Life Sciences, Northwest A&F University, No.22 Xinong Road, Yangling 712100, China; Heilongjiang Key Laboratory of Plant Bioactive Substance Biosynthesis and Utilization, Northeast Forestry University, No. 26, Hexing Road, Xiangfang District, Harbin 150040, China; College of Life Sciences, Northwest A&F University, No.22 Xinong Road, Yangling 712100, China; College of Life Sciences, Northwest A&F University, No.22 Xinong Road, Yangling 712100, China

## Abstract

The ubiquitin-26S proteasome system (UPS) is associated with protein stability and activity, regulation of hormone signaling, and the production of secondary metabolites in plants. Though the mechanism of action of SmMYB36 on the tanshinone and phenolic acid biosynthesis is well understood, its regulation through post-translational modifications is unclear. A constitutive photomorphogenesis 9 (COP9) signalosome subunit 5 (SmCSN5), which interacted with SmMYB36 and inhibited its ubiquitination-based degradation, was identified in *Salvia miltiorrhiza*. SmCSN5 promoted tanshinone biosynthesis but inhibited phenolic acid biosynthesis in the hairy roots of *S. miltiorrhiza*. SmMYB36 also activated the transcription of the target genes *SmDXS2* and *SmCPS1* but repressed that of *SmRAS* in a SmCSN5-dependent manner. SmCSN5 acts as a positive regulator in MeJA-induced biosynthesis of tanshinones and phenolic acids. Specifically, *SmCSN5* alone, when expressed transiently in tobacco and rice protoplasts, was localized to the cytoplasm, cell membrane, and nucleus, whereas when coexpressed with *SmMYB36*, it was detected only in the nucleus. Additionally, the degradation of SmMYB36^1–153^ by ubiquitination was lowered after truncation of the self-activating structural domain of SmMYB36^154–160^. Collectively, these results suggest that SmCSN5 affected the transcriptional activation of *SmMYB36* and stabilized SmMYB36, providing insights into the SmMYB36-based regulation of the accumulation of tanshinone and phenolic acid at the transcriptional and post-translational levels.

## Introduction

*Salvia miltiorrhiza* (‘red sage’) is a perennial herb of the Labiatae, native to China, and its dried roots and stems are a good source of phenolic acids and tanshinones [1]. It is effective in relieving swelling and pain, activating blood circulation, nourishing the heart, and calming the mind; and serves as the primary material for the treatment of myocardial infarction, coronary heart disease, hypertension, and hyperlipidemia [2–4]. The content of phenolic acids and tanshinones in natural resources is extremely low, and their accumulation is related to the geographical location, ecology, environment, and genetic background; and is regulated by inducers, crucial enzymes, and transcription factors (TFs) [5–7]. Due to clarity in the genomic background of *S. miltiorrhiza*, it has received increasing attention from researchers and is regarded as a ‘model medicinal plant’ for studying secondary metabolism synthesis and its regulation [8].

Tanshinones are synthesized primarily through the mevalonate (MVA) and the methylerythritol phosphate (MEP) pathways and phenolic acids by the Phe and Tyr pathways. Rosmarinic acid (RA) is a common precursor for the biosynthesis of most salvianolic compounds and is synthesized from Phe and Tyr via the activity of the enzymes such as phenylalanine ammonia-lyase (SmPAL), cinnidine-4-hydroxylase (SmC4H), 4-coumarate-CoA ligase (Sm4CL), tyrosine amino-transferase (SmTAT), hydroxyphenyl pyruvate reductase (SmHPPR), RA synthase (SmRAS), and cytochrome P450 (SmCYP98A14) [9, 10]. The MVA pathway starts with acetyl-CoA acetyltransferase (*SmAACT*) catalyzing the generation of acetoacetyl-CoA from acetyl-CoA, which is then combined with hydroxymethylglutaryl-CoA synthase (*SmHMGS*) to generate 3-hydroxy-3-methylglutaryl-CoA (HMG-CoA).

1-Deoxy-D-xylulose-5-phosphate synthase 2 (SmDXS2) catalyzes the generation of 1-deoxy-D-xylulose-5-phosphate (DXP) from glyceraldehyde-3-phosphate (GAP) and pyruvate as the first step in the MEP pathway [11]. The biosynthesis of tanshinone involves three stages: geranylgeranyl pyrophosphate (GGPP) synthesis, GGPP cyclization, and hypotanshinone diene modification. The synthesis of GGPP is similar to that of other natural diterpenoids. Isopentenyl pyrophosphate (IPP) is synthesized by the MVA and dimethylallyl pyrophosphate (DMAPP) by enzymes such as geranylgeranyl diphosphate synthase (SmGGPPS), farnesyl diphosphate synthase (SmFPPS), kaurene synthase-like 1 (SmKSL1), copalyl diphosphate synthase 1 (SmCPS1), and cytochrome P450-dependent monooxygenase (SmCYP76AH1, SmCYP76AH3, SmCYP76AK1, SmCYP76AH3, SmCYP71D375, and SmCYP71D373), and Fe(II)/2-oxoglutarate-dependent dioxygenase (Sm2OGD25) [1, 12, 13].

Many of the genes listed above have been shown to be regulated by TFs, which in turn are regulated by hormones such as jasmonic acid [14]. The insights into the transcriptional regulatory mechanisms involved in TFs are inadequate, and further studies are needed to understand the processes involved in the regulation by DNA methylation, microRNAs (miRNAs), and post-translational modifications [14]. MYB is one of the most prominent families of TFs in plants and can be classified into four major groups based on the number of repeats (R) they contain [15]. Those involved in the regulation of secondary metabolite synthesis mainly belong to the R2R3-MYB subfamily [7]. In *S. miltiorrhiza*, SmMYB1 [16], SmMYB2 [17], SmMYB39 [18], and SmMYB111 [19] regulate phenolic acid biosynthesis; SmMYB52 [20] regulates tanshinone biosynthesis; and SmMYB4 [21], SmMYB97 [22], and SmMYB98 [23] regulate the biosynthesis of both tanshinones and phenolic acids. These observations suggest that R2R3-MYB forms a network regulating the biosynthesis of phenolic acids and tanshinones at the transcriptional level, but the regulation at the post-translational level has not yet been reported.

The COP9 (constitutive photomorphogenesis 9) signalosome (CSN) demonstrated a CSN5-mediated deneddylation-modification activity on the E3 ubiquitin ligase complex, Cullin–RING ligase (CRL), facilitating the regulation of ubiquitination of the substrate proteins [24–26]. CSN consisted of eight subunits whose structures were similar to the components of the ‘lid’ of the 26S proteasome [27]. CSN5 and CSN6 possessed Mpr1p and Pad1pN-terminal (MPN) subunits; the two MPN structural domain proteins were mainly associated with the deubiquitination of the COP9 signalosome and were differentiated into the biochemically active MPN^+^ CSN5 and the inactive MPN^−^ CSN6 [28]. CSN5 participates in protein degradation as a CSN complex and is bound to target proteins as a monomer to regulate their stability [29, 30]. For example, the E3 ligase SCF^SLY1^ degraded the REPRESSOR OF ga1–3 LIKE 2 (RGL2), which was positively regulated by CSN; CSN5A, in a CSN complex-independent manner, promoted the degradation of the abscisic acid (ABA) INSENSITIVE 5 (ABI5) downstream of RGL2; thus facilitating crosstalk between ABA- and gibberellin (GA)-based signaling and the regulation of seed germination [30]. The involvement of CSN5 in the regulation of secondary metabolite biosynthesis in plants has recently been reported. In tomato, SlCSN5–2 promoted the ubiquitination degradation of SlBBX20 *in vivo*, thereby inhibiting the accumulation of anthocyanins [31].

We have recently identified a transcription factor SmMYB36 between SG5 and SG15, which regulates the biosynthesis of tanshinone and phenolic acids in *S. miltiorrhiza* [32]. Interestingly, SmMYB36 exerts opposite effects on the metabolic pathways with a positive effect on tanshinones and a negative on phenolic acids. SmMYB36 regulated the biosynthesis of phenolic acids and tanshinones three-dimensionally: affecting both the primary and secondary metabolic pathways, involving positive and negative regulation, and participating in direct and indirect regulation. SmMYB36 binds the promoters of *SmDXS2*, *SmGGPPS1*, and *SmCPS1*, which encode the enzymes in tanshinone biosynthesis, and promotes their expression. SmMYB36 also binds to the promoters of *SmGAPC*, encodinga 3-phosphoglyceraldehyde dehydrogenase, and of *SmRAS*, encoding an enzyme initiating phenolic acid biosynthesis, and represses their expression. Furthermore, MYB36 binds to the promoters of *SmERF6* and of *SmERF115*, which encode positive regulators of tanshinone and phenolic acid biosynthesis, respectively, promoting expression of the former and repressing the latter [32]. In conclusion, SmMYB36 was found to exert complex regulatory action on its downstream targets, thus providing a possibility for prioritizing one or the other pathway depending on the plant’s needs. However, whether and how SmMYB36 is itself regulated remained to be studied.

Herein, we report that SmMYB36 interacts with SmCSN5, a component of COP9 signalosome that targets proteins for degradation. Our results show that SmCSN5 enhances the stability SmMYB36, and is recruited to the nucleus when coexpressed with SmMYB36. SmCSN5 enhanced the binding of SmMYB36 to target genes in the hairy roots of *S. miltiorrhiza*, and the regulation of these genes by SmMYB36 was dependent on SmCSN5. In accordance with the previously reported regulatory functions of SmMYB36, SmCSN5 positively regulated the synthesis of tanshinones but negatively regulated the synthesis of phenolic acids. Therefore, the SmMYB36-SmCSN5 offers a module to differentially regulate the biosynthesis of phenolic acids and tanshinones in response to methyl jasmonate (MeJA).

## Results

### Overlapping ubiquitinated-degradation elements and transcriptional activation domains of SmMYB36

**Figure 1:**
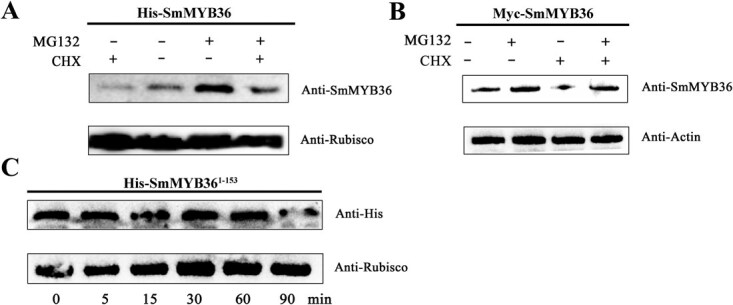
**Analysis of SmMYB36 degradation by proteasome/ubiquitination.** (**A**) Characterization of the *in vivo* ubiquitination-based degradation of SmMYB36 by the overexpression of *SmMYB36* in the tobacco transient expression system. Total protein was extracted from the tobacco leaves, which transiently expressed *SmMYB36*-*GFP*, treated with 0.1% DMSO, 100 μM CHX, 50 μM MG132, and 100 μM CHX + 50 μM MG132 for 12 h. Abundance of SmMYB36 was detected using anti-SmMYB36 antibody. RuBisCo was used as the loading control. (**B**) Overexpression of *SmMYB36* in Arabidopsis to characterize the ubiquitination-based degradation of SmMYB36 *in vivo*. Total protein was extracted from the *SmMYB36*-transgenic seedlings cultured normally on half-MS medium for 14 days treated with 0.1% DMSO, 100 μM CHX, 50 μM MG132, and 100 μM CHX + 50 μM MG132 for 6 h. The abundance of SmMYB36 was detected using anti-SmMYB36 antibody. Actin was used as the loading control. (**C**) The ubiquitination-based degradation of His-SmMYB36^1–153^ was characterized *in vitro* using a cell-free assay. The purified His-SmMYB36^1–153^ protein was added to a degradation reaction solution (prepared) containing 0.1% DMSO of total *S. miltiorrhiza* protein, and the abundance of His-SmMYB36 was measured using anti-SmMYB36 antibody at 0, 5, 15, 30, 60, and 90 min.

*In vivo*, assays were performed in exogenous tobacco and Arabidopsis plants to explore whether SmMYB36 was degraded via the 26S proteasome pathway. The total protein from *S. miltiorrhiza* was extracted for *in vitro* cell-free assays. The identification of the positive lines of Myc-SmMYB36-transgenic Arabidopsis plants by anti-Myc-based immunoblotting revealed no detectable signals in the WT plants, while bands corresponding to the size of the target proteins (M36O-1, 2, and 3) appeared with Myc-SmMYB36 (Fig. S2). Treatment with the proteosome inhibitor 50 μM MG132 enhanced the accumulation of SmMYB36-GFP in tobacco leaves and Myc-SmMYB36 in transgenic Arabidopsis plants compared with control (0.1% DMSO) (Fig. 1a–b). Compared with the control, treatment with 100 μM CHX inhibited the synthesis of SmMYB36-GFP/Myc-SmMYB36 protein, whose abundance appeared to be a synergistic effect of the two treatments after treatment with 50 μM MG132 + 100 μM CHX (Fig. 1a–b). These results demonstrated that SmMYB36 was degraded by the ubiquitination pathway.

A 12-amino-acid component of the MYC2 transcriptional activation domain, essential for protein hydrolysis and transcriptional activation, was identified [33]. A similar transcriptional activation element, SmMYB36^154–160^, was also identified [32]. To establish the relationship between the seven acidic amino acids ‘LELDEDE’ of SmMYB36 and proteolysis, the His-SmMYB36^1–153^ protein was obtained, and the total leaf protein of *S. miltiorrhiza* was extracted to perform a cell-free assay. SmMYB36^1–153^ showed no remarkable degradation at 5, 15, 30, 60, and 90 min compared to 0 min (Fig. 1c). These results indicate that SmMYB36^154–160^ is required for protein degradation and transcriptional activation.

### Sequence characterization and expression pattern analysis of SmCSN5

To further investigate the molecular mechanisms underlying the function of SmMYB36 at the post-translational level, the Y2H cDNA library was used to screen for SmCSN5, an interacting protein that may be involved in ubiquitination-based modification. The full-length ORF of *SmCSN5* consisted of 783 bp and encoded a protein, 261 amino acid-long, with a molecular weight of 29.07 kDa.

**Figure 2:**
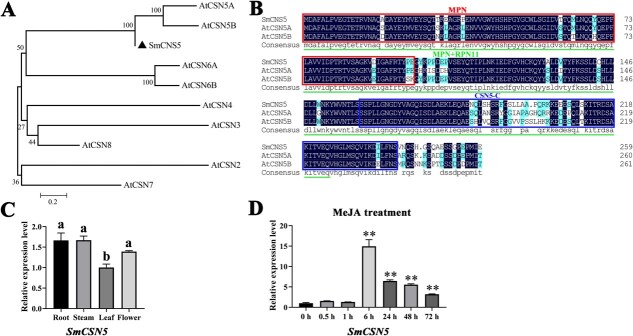
**Sequence characterization, phylogenetics, and analysis of the expression patterns of SmCSN5.** (**A**) SmCSN5 and CSNs from Arabidopsis were employed to construct a dendrogram of sequence similarity. Numbers on the branches are bootstrap values, based on the NJ method 1000 bootstrap. The bar represents 0.2. (**B**) Alignment of multiple amino acid sequences of SmCSN5 and AtCSN5A/B. TheN-terminal box box refers to the MPN structural domain, the sequence labeled by the green horizontal line to the MPN + RPN11 structural domain, and the other box to the C-terminus of CSN5. (**C**) Expression of *SmCSN5* in roots, stems, leaves, and flowers. The different letters: a, b, d, and c represent the statistical significance of the differences between the results, calculated using one-way analysis of variance (ANOVA) and Duncan’s test at *P* < 0.05. (**D**) Expression levels of *SmCSN5* at 0, 0.5, 1, 6, 24, 48, and 72 h after treatment with 100 μM MeJA. Error bars represent the standard deviation (SD) of three biological replicates. ** represent *P* < 0.05 and *P* < 0.01, respectively, calculated using the Student’s *t*-test.

To ascertain the protein sequence of SmCSN5 and analyze its evolutionary relationship, homologous proteins were selected for multiple amino acid sequence alignment, and a phylogenetic tree was established using the CSN1–8 subunits of Arabidopsis, respectively. SmCSN5 clustered in the same branch as AtCSN5A and AtCSN5B and also possessed the conserved domains of JAB/MPN (1–99), MPN/Rpn11 (1–224), and CSN5-C (159–239) (Fig. 2a–b).

For a deeper understanding of the expression patterns of *SmCSN5*, its tissue-specific (roots, stems, leaves, and flowers) and MeJA-induced expression were analyzed. On treatment with 100 μM MeJA, the expression levels of *SmCSN5* at 6, 24, 48, and 72 h were significantly higher than that of the control (0 h) and reached a peak of ~15-fold compared to the control at 6 h (Fig. 2c). Moreover, the expression levels were highest in the roots and stems but lowest in the leaves (Fig. 2d). These results suggest that SmCSN5 may be involved in the MeJA signaling pathway.

### SmCSN5 interacts with SmMYB36 *in vivo* and *in vitro*

**Figure 3:**
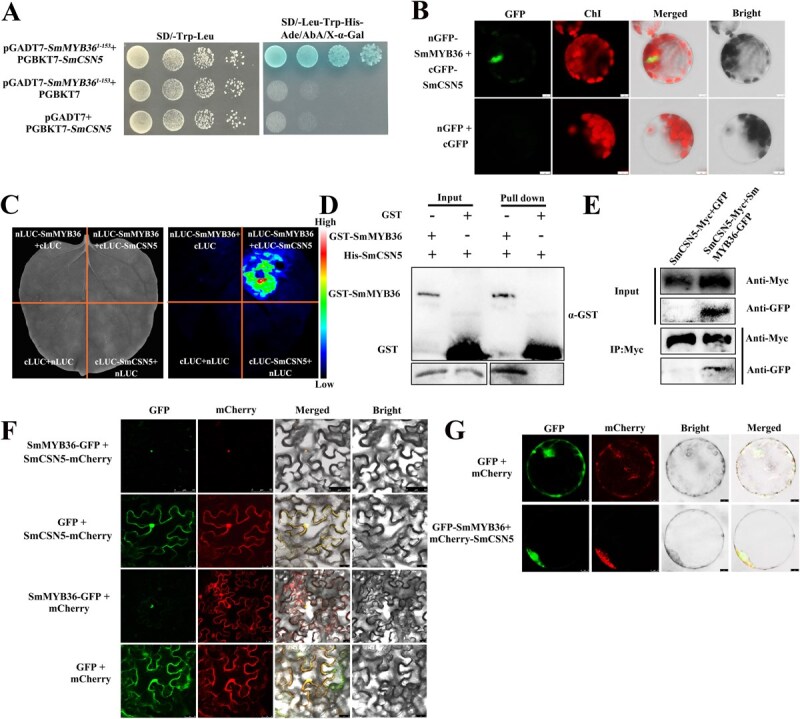
**Determination of the interaction between SmCSN5 and SmMYB36.** (A) Y2H assay employing SmCSN5 and SmMYB36. The Y2Hgold cells cotransformed with pGADT7-*SmMYB36*^1–153^ and pGBKT7-*SmCSN5* were incubated on SD/−Leu-Trp (left) and SD/−Leu-Trp-His-Ade/AbA/X-α-Gal (right) respectively, at 30°C for 3 days, and the growth was observed. (**B**) BiFC assay using SmCSN5 and SmMYB36. Arabidopsis protoplasts were cotransformed with constructs to express proteins in which SmMYB36 was fused to the N-terminal end of GFP (SmMYB36-nGFP) and SmCSN5 to the C-terminal end of cGFP (SmCSN5-cGFP), with cGFP + nGFP as a negative control. The standard bars represent 7.5 μm. (**C**) LCI assay employing SmCSN5 and SmMYB36. Plasmid combinations from the experimental group (nLUC-SmMYB36 + cLUC-SmCSN5) and the control groups (nLUC-SmMYB36 + cLUC, nLUC + cLUC-SmCSN5, and nLUC + cLUC) were used to infiltrate tobacco leaves. (**D**) Pull-down assay. SmMYB36 and SmCSN5 were fused to GST and HIS tags with molecular weights of ~34 and 40 kD, respectively. Anti-GST antibody was used to detect SmCSN5 and GST, and anti-His antibody to detect SmMYB36. (**E**) CoIP assay. SmMYB36 and SmCSN5 were fused with GFP and Myc tags, respectively, and expressed to set up an experimental (SmMYB36-GFP + SmCSN5-Myc) and a control combination (SmMYB36-GFP + Myc). Immunoprecipitation was performed using an anti-Myc antibody, and immunoblot analysis using anti-Myc and anti-GFP antibodies. (**F**) Colocalization analysis of SmCSN5 and SmMYB36 in tobacco transient transformation systems. SmCSN5 was fused with GFP and SmMYB36 with the mCherry tags and expressed. The experimental (SmMYB36-GFP + SmCSN5-mCherry) and control groups (SmMYB36-GFP + mCherry, GFP + SmCSN5-mCherry), scale bars represent 5 and 50 μm respectively. (**G**) Colocalization analysis of SmCSN5 and SmMYB36 in rice protoplasts. SmCSN5 and SmMYB36 were fusion-expressed with mCherry and GFP tags, respectively, and experimental (GFP-SmMYB36 + mCherry-SmCSN5) and control (GFP + mCherry) groups were set up to cotransform rice protoplasts. Scale bars represent 50 μm, respectively.

The prey vector pGADT7-*SmMYB36*^1–153^ and the bait vector pGBKT7-*SmCSN5* were constructed to further validate the experimental results of Y2H, with the results indicating an interaction between SmCSN5 and SmMYB36 (Fig. 3a). BiFC and LCI assays were performed to verify the interaction between SmMYB36 and SmCSN5 *in vivo*. SmMYB36 was fused at the N-terminal end of GFP and SmCSN5 to the C-terminal end. Fluorescence emitted by GFP was detected in Arabidopsis protoplasts expressing the nGFP-SmMYB36 + cGFP-SmCSN5 and nGFP + cGFP proteins. The results revealed that SmMYB36 interacted with SmCSN5 as the two fragments of GFP fluorescent were fused to form a complete GFP and green fluorescence was observed in the nucleus, while the control did not show any fluorescence (Fig. 3b). For the LCI experiments, SmMYB36 and SmCSN5 were ligated to the N- and C-terminal segments of Luciferase (LUC), respectively, and the tobacco transient expression system was used. The regions of the tobacco leaves expressing the experimental group (nLUC-SmMYB36 + cLUC-SmCSN5) indicated a strong fluorescence, while those expressing the control group (cLUC-SmCSN5 + nLUC, cLUC + nLUC-SmMYB36, and cLUC + nLUC) did not show any fluorescence. These results indicate that when heterologously expressed in tobacco, these two protein fragments of LUC interacted to bring the two fragments in close proximity and form a complete active enzyme, which reacted with the substrate to produce a fluorescent signal (Fig. 3c).

To confirm the *in vitro* interaction between SmMYB36 and SmCSN5, the His-SmMYB36 and GST-SmCSN5 proteins were purified through induction expression for the pull-down assay. His-SmMYB36 and GST-SmCSN5 were detected using anti-His after coincubation with GST-agarose gels, while no band of a corresponding size appeared in the negative control (His-SmMYB36 + GST) (Fig. 3d). Further, their interactions were determined using the CoIP assay. Total protein was extracted in tobacco leaves from the experimental group (SmCSN5-Myc + SmMYB36-GFP), and the negative control (SmCSN5-Myc + GFP) and immunoprecipitated with Myc antibody-conjugated protein A/G agarose. SmMYB36-GFP was detected by the Myc-antibody in the experimental group, while the negative control did not show any signal (Fig. 3e). Thus, these results demonstrated that SmCSN5 and SmMYB36 interact *in vivo* and *in vitro*.

### SmMYB36 relocalized the SmCSN5 from the cell membrane and cytoplasm to the nucleus

To ascertain whether SmMYB36 and SmCSN5 interacted and mutually affected localization, the plasmids SmMYB36-GFP and nuclear SmCSN5-mCherry expressing different fluorescent-tag proteins were constructed for colocalization assays in tobacco leaves. After the coexpression of different combinations of controls (SmMYB36-GFP + mCherry, GFP + SmCSN5-mCherry, and GFP + mCherry), SmMYB36-GFP was identified to be localized in the nucleus; GFP and mCherry in the whole cell; and SmCSN5-mCherry in the nucleus, cytoplasm, and cell membrane (Fig. 3f). However, SmMYB36-GFP and SmCSN5-mCherry were localized only to the nucleus after the coexpression of SmMYB36-GFP and SmCSN5-mCherry. In the same result, mCherry-SmCSN5 + GFP-SmMYB36 was transformed into rice protoplasts, and fluorescence was found only in the nucleus, while mCherry + GFP was localized throughout the cell. Hence, the results of the colocalization and BiFC experiments suggested that SmMYB36 interacted with SmCSN5, causing the relocalization of SmCSN5 from the cell membrane and cytoplasm to the nucleus.

### SmCSN5 promotes the accumulation of tanshinones and inhibits the accumulation of phenolic acids in the hairy roots of *S. miltiorrhiza*

**Figure 4:**
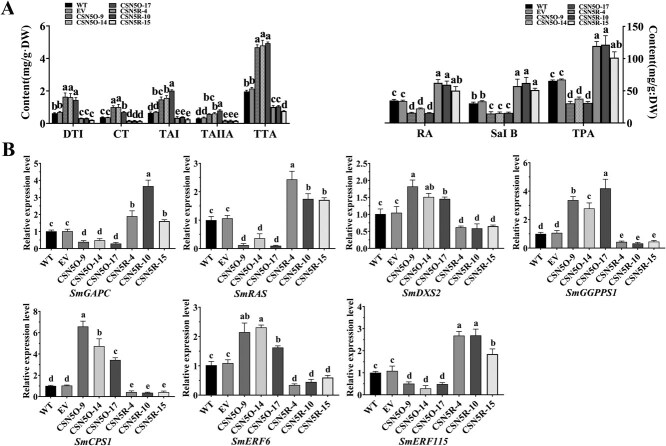
**Analysis of the effects of SmCSN5 on the biosynthesis of tanshinones and phenolic acids.** (**A**) HPLC-based determination of tanshinones (DTI, CT, TA1, and TAIIA) and phenolic acids (Sal B and RA) in the hairy roots of the experimental (CSN5O and CSN5R) and the control group plants (EV and WT). (**B**) The expression levels of *SmGAPC*, *SmRAS, SmDXS2*, *SmGGPPS1*, *SmCPS1*, *SmERF6*, and *SmERF115* in the hairy roots of the experimental (CSN5O and CSN5R) and control group plants (WT and EV) were determined using qRT-PCR. The different letters: a, b, c, and d represent the statistical significance of the differences in the results calculated using one-way ANOVA and Duncan’s test at *P* < 0.05.

The transgenic hairy root lines overexpressing *SmCSN5* (CSN5O-9, 14, and 17) and *SmCSN5*-RNAi (CSN5R-4, 10, and 15) were obtained to characterize the function of SmCSN5 in the hairy roots of *S. miltiorrhiza*. The empty vector (EV), transgenic, and RNAi lines demonstrated a typical expression of the red fluorescent protein, unlike the WT (the empty *Agrobacterium*) (Fig. S3a). The three lines: CSN5O-9, 14, and 17 the *SmCSN5* expression levels of 41.9-, 52.5-, and 73.8-fold higher than the WT; and the three lines: CSN5R-4, 10, and 15 with an *SmCSN5* expression level, 0.28-fold lower than that of the WT were selected for subsequent experiments; the expression of SmMYB36 was unchanged in both control and transgenic hairy root lines (Fig. S4a–b). The hairy roots of the transgenic lines were more profound in color than the controls (WT and EV), while the RNAi lines were lighter (Fig. S3b). HPLC detection indicated that the contents of the tanshinones: DTI, CT, TAI, TAIIA, and TTA were remarkably higher in the *SmCSN5*-overexpressing lines, whereas were markedly lower in the *SmCSN5*-RNAi lines compared to the controls (Fig. 4a). Inversely, the overexpression lines were conspicuously lower in the phenolic acids RA, Sal B, and TPA, but significantly higher in the RNAi lines compared to the controls (Fig. 4a). Meanwhile, the expression levels of the target genes of SmMYB36: *SmGAPC*, *SmRAS*, *SmDXS2*, *SmGGPPS1*, *SmCPS1*, *SmERF6*, and *SmERF115* in the *SmCSN5-*overexpressing hairy root lines were detected by qRT-PCR. The expression levels of the genes: *SmDXS2*, *SmGGPPS1*, and *SmCPS1,* encoding the tanshinone synthesis-related enzymes, and *SmERF6,* encoding a positive regulator, were significantly upregulated; while *SmRAS* encoding a phenolic acid synthesis-related enzyme and SmERF115 encoding a positive regulator were remarkably downregulated; however, the opposite results were observed in the RNAi-hairy root lines (Fig. 4b), *SmGAPC*, encoding a primary enzyme was markedly downregulated in the overexpressing lines but upregulated in the RNAi lines (Fig. 4b). These results therefore suggested that SmCSN5 promoted the accumulation of tanshinones but inhibited that of phenolic acids.

### SmCSN5 positively regulates MeJA-induced tanshinone biosynthesis and negatively regulates MeJA-induced phenolic acid biosynthesis.

**Figure 5:**
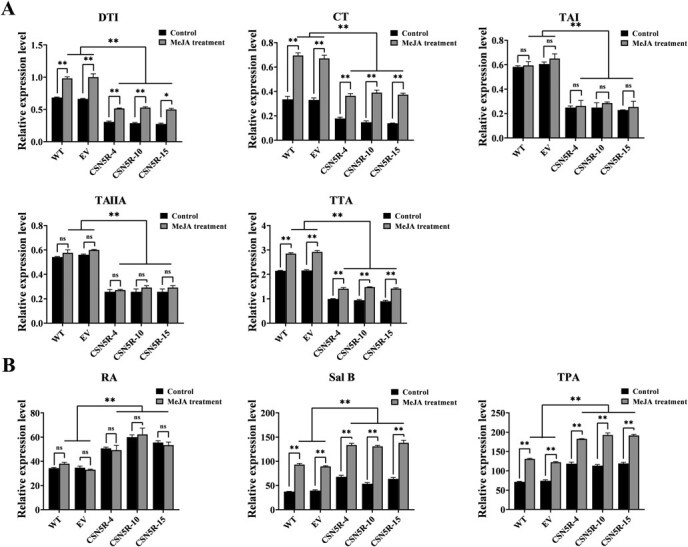
Function of SmCSN5 in MeJA-induced phenolic acid and tanshinone biosynthesis in *S. miltiorrhiza* hairy root. (**A–B**) Changes in tanshinone and phenolic acid content in 100 μM MeJA-treated and untreated 7-day *SmCSN5*-RNAi transgenic hairy root lines and control (WT and EV). Error bars indicate the SD of the three biological replicates. ** represents *P* < 0.01, respectively, calculated using the Student’s *t*-test.

To clarify the effects of MeJA on tanshinone and phenolic acid biosynthesis and the relationship with the regulatory functions of SmCSN5, the contents of tanshinones and phenolic acids were determined in 100 μM MeJA-treated and untreated 7-day SmCSN5-RNAi transgenic hairy root lines (CSN5R-4, 10, and 15) and in controls (WT and EV). As the results show in Fig. 5, after MeJA treatment, the DTI, CT, and TTA contents in WT and EV were significantly increased, with DTI and CT contents increasing by 0.297–0.337 and 0.340–0.360 mg/g, respectively; DTI, CT, and TTA contents in CSN5R-4, 10, and 15 were also significantly induced by MeJA, with DTI contents increasing by 67.7%, 84.0%, and 82.0%, and CT content increased to 2.04, 2.65, and 2.68 times of the untreated levels, while TAI and TAIIA content did not change significantly; DTI, CT, TAI, TAIIA, and TTA contents were significantly higher in WT and EV than in CSN5R-4, 10, and 15; and the mean increment of TTA content was significantly higher in WT and EV than the mean increment in CSN5R-4, 10, and 15 mean increments. Concurrently, Sal B and TPA contents were significantly upregulated in both control and RNAi lines after MeJA treatment, and Sal B and TPA contents were significantly higher in RNAi lines than in the control, and the mean increase in Sal B content in RNAi strains was significantly greater than that in controls. The above results indicated that MeJA induced the biosynthesis of tanshinones (DTI and CT) and salvianolic acid (Sal B); SmCSN5 is a positive regulator of MeJA-induced tanshinone biosynthesis and a negative regulator in MeJA-induced phenolic acid biosynthesis; and the MeJA treatment could not backfill the loss of function of SmCSN5.

### Regulation of phenolic acid and tanshinone biosynthesis by SmMYB36 was dependent on SmCSN5

**Figure 6:**
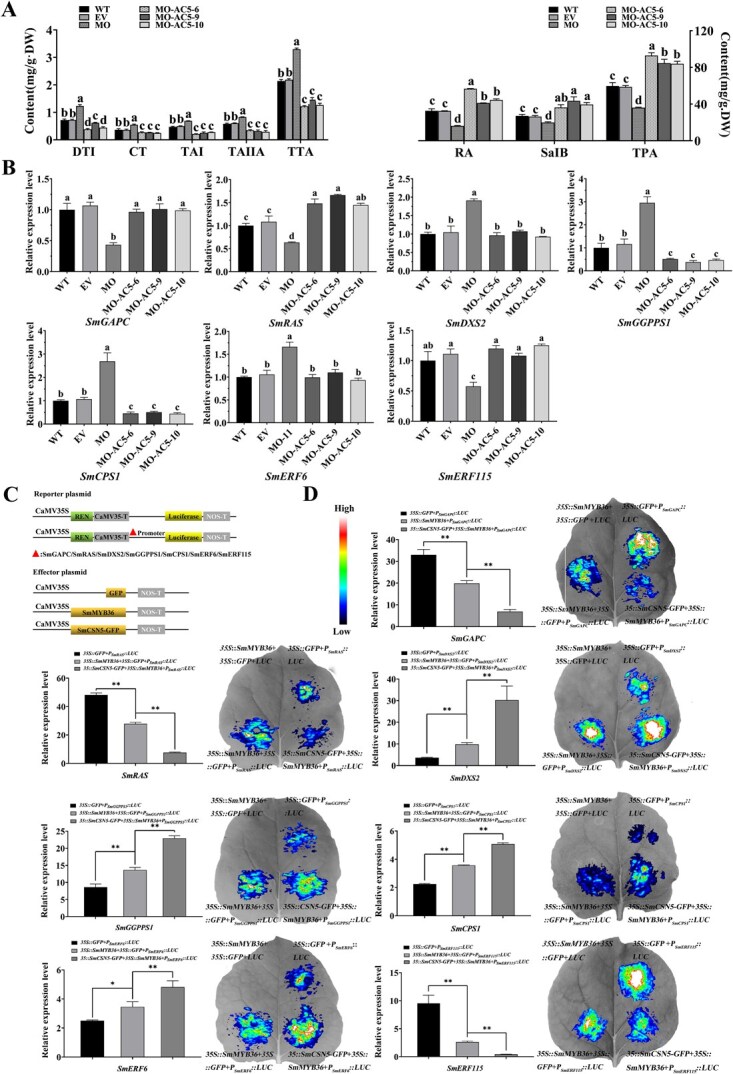
**Whether the role of SmMYB36 in promoting tanshinone biosynthesis but inhibiting phenolic acid biosynthesis is dependent on the analysis of SmCSN5.** (**A**) HPLC-based determination of tanshinones (DTI, CT, TAI, and TAIIA) and phenolic acids (Sal B and RA) in the hairy roots of the experimental (MO and MO-CA) and control group plants (WT and EV). (**B**) The expression levels of *SmGAPC*, *SmRAS*, *SmDXS2*, *SmGGPPS1*, *SmCPS1*, *SmERF6*, and *SmERF115* in the hairy roots of the experimental (MO and MO-CA) and control group plants (WT and EV) were determined using qRT-PCR. Error bars represent SD (*n* = 3). *SmActin* was used as an internal reference. The different letters: a, b, c, and d represent the statistical significance of the differences in the results calculated using one-way ANOVA and Duncan’s test at *P* < 0.05. (C) Schematic diagram indicating the construction of the reporter and effector plasmids. (D) The combinations of the reporter plasmids (*P_SmGAPC_*::*LUC*, *P_SmRAS_*::*LUC*, *P_SmDXS2_*::LUC, *P_SmGGPPS1_*::*LUC*, *P_SmCPS1_*::*LUC*, *P_SmERF6_*::*LUC*, and *P_SmERF115_*::LUC) with the effector plasmids (*GFP*, *SmMYB36*, and *SmMYB36 + SmCSN5*-*GFP*) were coinjected to measure the LUC activity in tobacco leaves, respectively. The LUC activity values of the reporter and the *SmMYB36* plasmid combinations were used as controls. The error bars indicate the SD of the three biological replicates. * and ** indicate *P* < 0.05 and *P* < 0.01, respectively, calculated using Student’s *t*-test.

Transgenic hairy roots with *SmMYB36*-overexpression alone (MO) or *SmMYB36*-overexpression with *SmCSN5*-antisense expression (MO-AC5) were obtained to determine the role of SmCSN5 in the regulation of tanshinone and phenolic acid biosynthesis by SmMYB36. The MO; MO-AC5–6, 9, and 10; and the EV-hairy root lines expressed the red fluorescent protein, not the WT (Fig. S5a). qRT-PCR demonstrated that the expression levels of *SmMYB36* in MO were at least 40-fold higher compared to WT, whereas those of *SmCSN5* did not change significantly compared to the controls (EV and WT) (Fig. S6a). In the MO-AC5–6, 9, and 10 lines, the expression of *SmMYB36* was upregulated by ~18-, 28-, and 29-fold, respectively; while *SmCSN5* was downregulated by ~0.30-, 0.34-, and 0.24-fold, respectively, compared to the WT (Fig. S6b). The hairy root phenotype of MO was markedly darker than those of MO-AC5–6, 9, and 10 (Fig. S5b). The overexpression of *SmMYB36* remarkably upregulated the contents of the tanshinones: DTI, CT, TAI, TIIA, and TTA; but markedly downregulated those of the phenolic acids: RA, Sal B, and TPA compared to the control (Fig. 6a). Conversely, in the lines MO-AC5–6, 9, and 10, the contents of DTI, CT, TAI, and TIIA decreased conspicuously, with 44.4%, 33.4%, and 42.3% decrease in TTA; while those of Sal B and RA enhanced markedly, especially the TPA content being >1.41-fold that of the WT (Fig. 6a). Compared with MO, the lines MO-AC5–6, 9, and 10 showed a significantly lower content of DTI, CT, TAI, and TIIA, with that of TTA being at least 46% lower; while the contents of Sal B, RA, and TPA were significantly higher by ~2.58-, 2.34-, and 2.33-fold, respectively. However, in the MO-AC5–6, 9, and 10 lines, when compared with MO, the expression levels of *SmDXS2*, *SmGGPPS1*, and *SmCPS1,* which encode the enzymes related to tanshinone biosynthesis and *SmERF6* that encodes their positive regulator, were significantly downregulated; while those of *SmRAS*, which encoded the enzyme associated with the biosynthesis of phenolic acid, and *SmERF115*, which encoded its positive regulator, and *SmGAPC* were markedly up and downregulated, respectively, (Fig. 6b). Notably, the expression levels of *SmGGPPS1* and *SmCPS1* were markedly downregulated and that of *SmRAS* was conspicuously upregulated, while those of *SmDXS2*, *SmERF6,* and *SmERF115* did not change significantly in the lines MO-AC5–6, 9, and 10 compared to the WT (Fig. 6b). These results indicated that the regulation of tanshinone and phenolic acid biosynthesis by SmMYB36 was dependent on SmCSN5.

### SmCSN5 enhances the transcriptional regulation activities of SmMYB36

Dual-LUC assays were performed to investigate whether SmCSN5 affected the transcriptional regulatory effects of SmMYB36 on the target genes. Compared with the coexpression of the empty vector (GFP) and the reporter plasmid, SmMYB36 markedly enhanced the LUC activity of *P_SmDXS2_*::*LUC*, *P_SmGGPPS1_*::*LUC*, *P_SmCPS1_*::*LUC*, and *P_SmERF6_*::*LUC*, but markedly inhibited that of *P_SmGAPC_*::*LUC*, *P_SmRAS_*::*LUC*, and *P_SmERF115_*::*LUC* (Fig. 6d). In parallel, compared to the coexpression of the SmMYB36 + GFP and reporter plasmids, SmCSN5 markedly enhanced the expression of *P_SmDXS2_*::*LUC*, *P_SmCPS1_*::*LUC*, *P_SmGGPPS1_*::*LUC*, and *P_SmERF6_*::*LUC*, but remarkably inhibited that of *P_SmGAPC_*::*LUC*, *P_SmRAS_*::*LUC*, and *P_SmERF115_*::*LUC* (Fig. 6d). These results tentatively suggest that SmCSN5 promoted the biosynthesis of tanshinones but inhibited that of phenolic acids by enhancing the regulatory effects of SmMYB36 on target genes.

### SmCSN5 enhances the stability of SmMYB36

**Figure 7:**
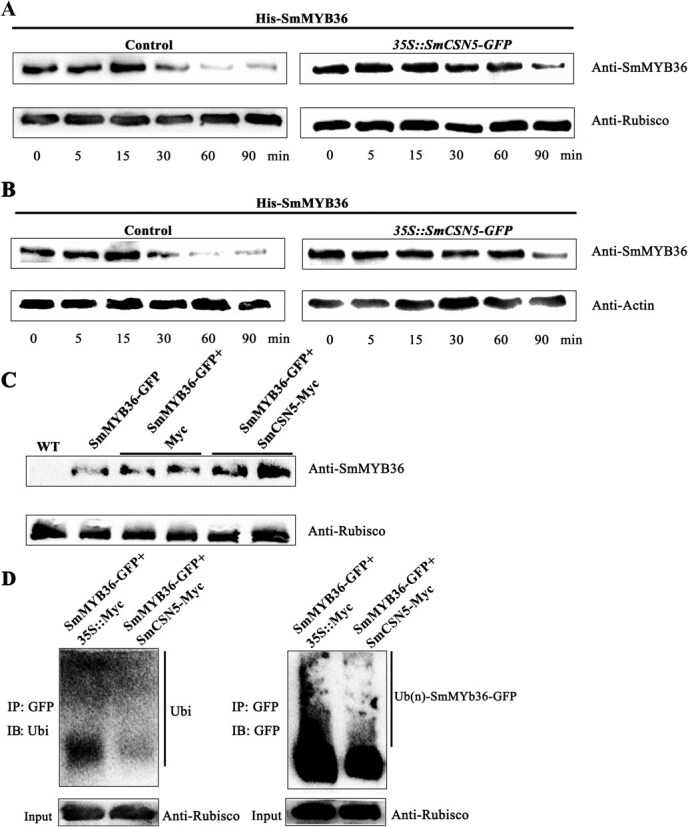
**Effect of SmCSN5 on the ubiquitination-based degradation of SmMYB36.** (**A**) The inhibitory effect of SmCSN5 on the ubiquitination-based degradation of SmMYB36 was ascertained using an *in vitro* cell-free assay. The total protein obtained from tobacco leaves overexpressing *SmCSN5* was added to the degradation buffer. The purified His-SmMYB36 protein was added to the degradation reaction solution in the experimental group (overexpressing *SmCSN5*-Myc) and the control group (overexpressing Myc). The abundance of His-SmMYB36 at 0, 5, 15, 30, 60, and 90 min was measured using the anti-SmMYB36 antibody. (**B**) The repressive effect of SmCSN5 on the ubiquitination-based degradation of SmMYB36 was characterized *in vitro* using the total protein extracted from the *SmCSN5*-transgenic Arabidopsis plants to formulate the degradation buffer. The purified His-SmMYB36 protein was added to the degradation reaction solution in the experimental group (*SmCSN5-GFP* overexpression) and the control group (WT). The abundance of His-SmMYB36 at 0, 5, 15, 30, 60, and 90 min was measured using anti-SmMYB36 antibody. (**C**) Coexpression of *SmMYB36* and *SmCSN5* in tobacco leaves was used to identify the inhibition of the ubiquitination-based degradation of SmMYB36 by SmCSN5 *in vivo*. Total proteins were extracted from the plants of the experimental (SmCSN5-Myc + SmMYB36-GFP) and control (Myc + SmMYB36-GFP and WT) group plants, and the abundance of SmMYB36-GFP was measured using anti-GFP antibody. (**D**) *SmMYB36* and *SmCSN5* were coexpressed in tobacco leaves, and the inhibition of the ubiquitination-based degradation of SmMYB36 by SmCSN5 *in vivo* was identified using CoIP assay. Total protein was extracted from the plants of the experimental group (SmCSN5-Myc + SmMYB36-GFP) and the control group (Myc + SmMYB36-GFP). The SmMYB36-GFP protein was immunoprecipitated with the anti-GFP antibody. Anti-ubiquitin antibodies were used to detect the ubiquitinated SmMYB36-GFP.

The recombinant His-SmMYB36 protein was used for *in vitro* cell-free experiments, and *in vivo* assays were performed using coinjected tobacco leaves to determine whether SmCSN5 affected the ubiquitination-based degradation of SmMYB36. *SmCSN5*-*GFP*-transgenic Arabidopsis plants were positively identified by anti-GFP immunoblotting, and no signal was detected in the WT plants, while the *SmCSN5*-*GFP* lines C5O-1, 2, and 3 demonstrated target-sized bands (Fig. S7). For cell-free experiments, the western blot signal of His-SmMYB36 was more potent at 30, 60, and 90 min compared to the control (WT) plants using the total protein extracted from the *SmCSN5*-*GFP* transgenic Arabidopsis plants. Likewise, the abundance of the His-SmMYB36 protein at 30, 60, and 90 min was also higher compared to the control (EV) plants using the total protein extracted from tobacco leaves transiently overexpressing *SmCSN5*-Myc (Fig. 7a–b). Additionally, total proteins were extracted from the tobacco leaves coexpressing SmMYB36-GFP + Myc and SmMYB36-GFP + SmCSN5-Myc, respectively, and detected using anti-SmMYB36 antibody; while SmMYB36-GFP was immunoprecipitated, and the ubiquitination level of SmMYB36 was detected using the anti-GFP and anti-Ubi antibodies. The signal of the western blot was more robust in the experimental group (SmMYB36-GFP + SmCSN5-Myc) than the control group (SmMYB36-GFP + Myc), and the ubiquitination level was higher after IP (Fig. 7c–d). Consequently, these results suggested that SmCSN5 inhibited the degradation or ubiquitination of SmMYB36 via the proteasome pathway.

## Discussion

### Ubiquitination of SmMYB36 regulates the biosynthesis of tanshinone and phenolic acid

It is well established that gene expression is generally regulated at transcriptional and translational levels. As proteins play prominent roles in cellular functions, they are the main effectors ultimately involved in biological processes. It is a complex and delicate process from RNA to protein to phenotype. After the transcription of mRNA, exemplary regulatory processes such as post-transcriptional alterations, epigenetic modifications (DNA methylation, etc.), and post-translational modifications (protein degradation and export) occur, and the abundance of mRNAs and proteins is likely inconsistent. SmMYB36 was observed to be degraded via the ubiquitination pathway through *in vitro* and *in vivo* experiments. To more deeply understand whether the degradation was fine-tuned through post-translational modifications, the Y2H library was used to identify SmCSN5, a protein that may affect the ubiquitination of SmMYB36. qRT-PCR revealed that the transcript levels of *SmMYB36* did not change significantly in the *SmCSN5*-overexpression and -RNAi hairy roots (Fig. S4b), while those of the target genes of SmMYB36 changed remarkably (Fig. 4b). This suggests that SmCSN5 regulated the expression of target genes by modulating the protein levels of SmMYB36.

Ubiquitin proteasome system (UPS)-mediated proteolysis is one of the crucial post-translational mechanisms regulating the transcriptional activation of TFs in higher plants. The most direct evidence is the occurrence of a 12-amino acid element in the transcription activation domain (TAD) of the Arabidopsis MYC2, which played a dual role as a signal for proteolysis and as a regulator of the transcriptional activity of MYC2 [33]. In response to ABA, the MYB30-INTERACTING E3 LIGASE 1 (MIEL1) mediated the degradation of MYB30 and inhibited the interaction of MYB30 with ABI5; MYB30 directly inhibited the transcriptional activation capacity of ABI5 on the ABA-responsive target genes to limit the ABA-mediated response [34]. Analogously, the elevated abundance of the RING finger E3 ligases MaBRG2/3 protein, which ubiquitinated and targeted MaMYB4 for degradation after the onset of the ripening process, attenuated the transcription of the MaMYB4-target genes, thereby delaying or promoting fruit ripening by repressing or stimulating ethylene biosynthesis [35]. These studies suggest that E3 ligases, directly and indirectly, regulated the transcriptional levels and post-translational modifications of TFs. Further, the effect of ubiquitination on the transcriptional activation of TFs was not limited to the E3 ligase, which played a direct role. The results of this study suggested that SmCSN5 promoted the stability of SmMYB36 and enhanced the effects of SmMYB36 on the transcription of target genes. Similarly, in apple, the BTB and TAZ structural domain protein 2 (MdBT2) mediated the ubiquitination-based degradation of AUXIN RESPONSE FACTOR 8 (MdARF8), accompanied by the repression of the transcription of the target gene of MdARF8, GRETCHEN HAGEN 3 (*MdGH3*.*1*/*3*.*6*), thereby negatively regulating the formation of adventitious roots [36]. Intriguingly, the inhibition of the expression of *SmCSN5* restored the abundance of the transcripts of a subset of target genes of SmMYB36 (*SmGAPC*, *SmDXS2*, *SmERF6*, and *SmERF115*) to be insignificantly different from that of the WT, unlike the other subset (*SmGGPPS1*, *SmCPS1*, and *SmRAS*) (Fig. 6b). These results suggest that the regulation of tanshinone and phenolic acid biosynthesis by SmMYB36 was dependent on SmCSN5 and that SmCSN5 regulated the expression of *SmRAS*, *SmGGPPS1*, and *SmCPS1* by affecting the transcriptional activity of the other regulatory factors.

Ubiquitination-based modifications of TFs regulating the biosynthesis of terpenoids and phenylpropanes have been extensively reported. For instance, SlBBX20 activated the expression of PHYTOENE SYNTHASE 1 (*PSY1*) to promote the accumulation of carotenoids, subject to the ubiquitination-associated degradation mediated by the CRL4E3 complex [37]. PHOSPHATE STARVATION RESPONSE1 (MdPHR1) promoted anthocyanin biosynthesis and was negatively regulated by the E3 ubiquitin ligase SEVEN IN ABSENTIA1 (MdSINA1) through ubiquitination-mediated regulation [38]. SmCSN5 regulated the terpenoids (tanshinones) and phenylpropanes (phenolic acids) biosynthesis in parallel with its precise control of the stability of SmMYB36. Conversely, the Kelch repeat F-box (KFB) protein, SmKFB5, negatively regulated phenolic acid synthesis through the ubiquitination-26S proteasome pathway, consequently mediating the degradation of the Phe aminolysis enzymes, PAL1/2/3. As a consequence, the regulation of the ubiquitination process for the production of secondary metabolites occurs in two major ways: by either directly affecting the stability of the enzymes, or indirectly through the ubiquitination-based degradation of TFs that modulate the abundance of the transcripts of the target genes.

### SmMYB36 facilitates the nuclear localization of SmCSN5

Light and JA signaling have been identified using a TF-dependent nuclear localization mechanism in Arabidopsis. The translocation of phytochromes A and B to the nucleus required their interaction with facilitators carrying the nuclear localization signals (NLS) in the cytoplasm [39]. The Jas motif of the JAZ9 protein was critical for the interaction with MYC2, nuclear localization, and whether it is degraded by SCF^COI1^, and the entry of JAZ9 into the nucleus was dependent on MYC2 [40]. Similar studies were performed with CSNs, where CONSTITUTIVE PHOTOMORPHOGENIC1 (COP1) responded to light through the coordinated action of the nuclear and cytoplasmic localization signals, and the N-terminal structural domain of CSN1 was required for the nuclear localization of COP1 in the hypocotyl cells of Arabidopsis [41]. In the darkness, COP1 was translocated to the nucleus in a CSN-dependent manner, where it interacted with the elongated hypocotyl-5 (HY5) and marked the HY5 for 26S proteasome-mediated degradation. Notably, SmMYB36 promoted the nuclear accumulation of SmCSN5 (Fig. 3b, f–g), while the degradation of SmMYB36 was inhibited (Fig. 7). These findings suggested an effect of the ubiquitination of TFs, and hence the regulation of gene transcription, through the dependence of the nuclear localization of the E3 ligase on CSN and the dependence of the nuclear localization of CSN on the targeted TFs. Additionally, several other studies have indicated that nuclear localization was closely related to ubiquitination-based modifications. For example, COP1, a positive regulator of ABA-related responses during the growth of Arabidopsis seedlings in the dark, promoted the stability of ABI5 by targeting the ubiquitinated-based degradation of the ABA-hypersensitive DCAF1 (ABD1**)** and ABA-related signaling also induced the nuclear accumulation of COP1 under dark, thereby enhancing the activity of ABA-related signaling *[**42**]*. BROAD-COMPLEX, TRAMTRACK, and BRIC A BRAC2 (*MdBT2*) interacted with MdCOP1, enhanced the abundance of MdCOP1 in the nucleus, and inhibited self-ubiquitination to stabilize MdCOP1, resulting in the reduction of anthocyanin biosynthesis [43]. Under the conditions of drought-induced stress, the SUMO E3 ligase protein, CaDSIZ1, accumulated in the nucleus, where it SUMOylated the TF, CaDRHB1, thereby protecting it from the ubiquitination-based degradation, and finally promoted the expression of drought-responsive genes [44]. A rapid and precise response or the inhibition of cellular processes by phytohormones or other signaling pathways is ensured by certain interdependent nuclear inputs, mainly involving protein–protein interactions, nuclear localization, and protein degradation.

**Figure 8:**
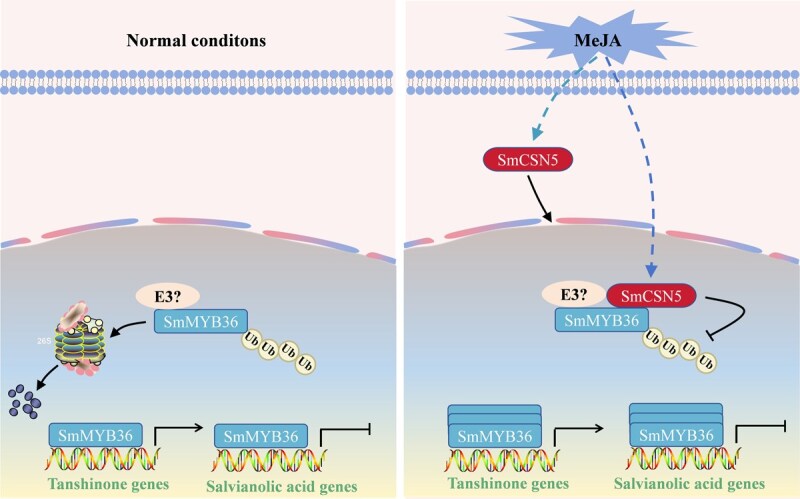
**A hypothetical working model explaining the SmCSN5**-**SmMYB36**-**based regulation of tanshinone and phenolic acid biosynthesis.** SmCSN5 interacted with SmMYB36 in the nucleus to promote the stability of SmMYB36 and enhance its ability to regulate the transcription of the target genes. MeJA promoted the expression of *SmCSN5*, thereby strengthening the capacity of SmMYB36 to regulate the transcription of the target genes, which led to a further improvement in tanshinone accumulation but a reduction in phenolic acid accumulation.

### MeJA-induced expression of *SmCSN5* enhances the stability of SmMYB36

Some studies have demonstrated the involvement of CSN5 in the biotic- and abiotic-stress resistance and hormone-based signaling in plants through the regulation of the cullin-RING ligase (CRL) and the UPS [45, 46]. For example, the interaction of the Cys2/His2-type zinc finger protein, SlZF3, with CSN5B may inhibit the degradation of GDP-Man pyrophosphatase 1 (VTC1) by the 26S proteasome pathway during stress, thus maintaining the ascorbic acid (AsA) content at a higher level, thereby scavenging the excess H_2_O_2_ and boosting the tolerance to various abiotic stresses [47]. Barley yellow striate mosaic virus (BYSMV) P6 protein negatively affected the CSN5-mediated de-RUBylation of Cullin1 (CUL1) by interacting with CSN5, resulting in the inability of the CUL1-based Skp1/Cullin1/F-box ubiquitin E3 ligase to mediate the degradation of the jasmonate ZIM-structure protein, leading to impaired JA signaling, and enhanced the attraction of insects to barley plants [48]. In *CSN*-silenced plants, the curtailment of the wound response corresponded to a decline in JA synthesis, but the levels of salicylic acid (SA) were unaltered [49]. Notably, the biosynthesis of phenolic acids and tanshinones was induced by the biotic- and abiotic-stress-associated inducers such as YE (yeast extract), Ag^+^, GA, ABA, SA, MeJA, and ethylene (Eth), through the activation or repression of the expression of essential genes encoding enzymes that play a role in the biosynthetic pathway with or without the involvement of different TFs to regulate the biosynthesis of secondary metabolites [50, 51]. Jasmonates (JAs) promoted the synthesis of phenolic acids and tanshinones by regulating the corresponding TFs associated with the JA-signaling pathway [19], explained by the core model of the SCF^COI1^-JAZs-MYC2 complex. Our results revealed that MeJA induced the biosynthesis of tanshinones (DTI and CT) and phenolic acid (Sal B) (Fig. 5). It was concluded that MeJA mainly induced the promotion of the synthesis of DTI and CT, whereas it had no significant or weak effect on the promotion of TAI and TAIIA accumulation, which is more in line with the results in this study [52]. Additionally, it has been found that MeJA as an inducer significantly increased the content of Sal B in the hairy roots of *S miltiorrhiza*, but the promotion effect on the accumulation of rosmarinic acid was unstable, probably because RA is the synthesis precursor substance for derivatives such as Sal B, and a large amount of RA was consumed by MeJA to promote the accumulation of downstream salvianolic acid [53]. PEG upregulated the expression of the tanshinone biosynthesis-related genes, *SmHMGR* and *SmDXS*, by triggering bursts in the reactive oxygen species (ROS) in the hairy roots of *S. miltiorrhiza* to enhance the production of tanshinones [54]. Optimal concentrations of SA promoted the accumulation of phenolic acids by enhancing the activity of PAL [55]. Our results indicate that the expression of *SmCSN5* was induced by the external factor MeJA (Fig. 2d), which positively regulated tanshinone and negatively regulated phenolic acid (Fig. 4). In tomato, SlMYB13 improves drought tolerance by enhancing scavenging of reactive oxygen species through increased phenolamide content and increased abscisic acid content [56]. These evidences led to the hypothesis that SmCSN5 is cross-regulated by multiple hormones and stress-related signaling pathways to achieve pinpoint regulation of tanshinone and phenolic acid biosynthesis.

The current understanding of the specific role of CSN in the ubiquitination process remains fragmented. In plants, CSN5 primarily acts as a facilitator [29, 31, 57]. The process of deneddylation occurs by the reversal of the NEDD8 (RUB1) modification on the Cullin subunit, and CSN enhanced the function of CRL and promoted the ubiquitination of target proteins [58, 59]. Interestingly, the present study revealed that SmCSN5 exerted a negative regulatory effect on the ubiquitination of SmMYB36. Similarly, CSN5 interacted with the E3 ligase Cullin 1 to inhibit the degradation of ‘RESISTANCE TO *PSEUDOMONAS SYRINGAE* 2’ (AtRPS2) and activate the effector-triggered immunity (ETI) response [60]. The function of the CSN complex in post-translational modifications (PTMs) is limited to deneddylation, phosphorylation, and deubiquitination [24]. CSN5 activated the phosphokinases [61] and deubiquitinated the target proteins [62] in mammals. A feedback regulation between the different types of modifications affected the protein stability. The stability of the E3 ubiquitin ligase XB3 ortholog 5 in *Arabidopsis thaliana* (XBAT35.2) and its substrate, the vacuolar protein-sorting 23A (VPS23A) were deubiquitinated by ubiquitin-specific protease 12 (UBP12) and UBP13; the competitive binding of XBAT35.2, UBP12, and 13 to VPS23A precisely regulated the ubiquitination-based degradation [63]. As such, three hypotheses are proposed to explain this phenomenon: the first possibility suggests that SmCSN5 enhanced the deubiquitination efficiency of SmMYB36 thereby improving stability; the second possibility is that either SmCSN5 competed with the E3 ligase for binding to SmMYB36 or reduced the interaction between the two, thereby inhibiting the ubiquitination-based degradation of SmMYB36; and the third possibility is that SmCSN5 negatively affected the E3 ligase acting on SmMYB36, and exercises deneddylation of SmMYB36, while SmCSN5 competed with E3 ligase for interacting with SmMYB36, thereby forming a positive feedback regulatory loop for SmMYB36. Nevertheless, SmCSN5 regulated the ubiquitination of SmMYB36, and the underlying mechanisms of action need to be explored further in detail.

### The SmCSN5-SmMYB36 module promotes the accumulation of tanshinones and inhibits the accumulation of phenolic acids

In recent years, the nuclear, mitochondrial, and chloroplast genomes of *S. miltiorrhiza* have been sequenced and explored [64, 65]. More than 20 publications are available using the transcriptomic data from different organs and growth stages of *S. miltiorrhiza* [66–68]. Most genes encoding crucial enzymes have been cloned and identified, effectively improving the contents of tanshinones and phenolic acids [9]. These findings provide an essential basis for elucidating the biosynthetic pathways of tanshinones and phenolic acids and revealing the mechanisms of their individual or cooperative regulation by TFs. A study identified 110 R2R3-MYB TFs from the whole genome of *S. miltiorrhiza*, classified them into 37 SGs, and predicted that the subgroups SG3, 4, 5, 6, 7, 13, 20, and 21 regulated the biosynthesis of tanshinones and phenolic acids [69], but the regulation at the level of post-translational modifications has not yet been understood. Several studies have reported that PTMs affect the synthesis of tanshinone and phenolic acids. For instance, the interaction of sucrose non-fermenting-1-related protein kinase 2 (SmSnRK2.6) with the ABA-responsive element (ABRE)-binding protein (SmAREB1) promoted the biosynthesis of phenolic acids [70]. MAPKK2/4/5/7-SmMAPK3-SmJAZ formed a cascade that regulated the accumulation of phenolic acids [71]. Therefore, this study fills the gap in understanding the regulation of the biosynthesis of phenolic acids and tanshinones by MYB TFs at the post-transcriptional level.

A model for the mechanism of action of SmCSN5-SmMYB36-target genes-based regulation of phenolic acid and tanshinone biosynthesis was proposed in combination with the previous findings of our group (Fig. 8). SmMYB36 activated the expression of the gene encoding a branching enzyme involved in tanshinone biosynthesis but inhibited the expression of that involved in the SA biosynthesis, thereby promoting tanshinone and inhibiting SA biosynthesis. MeJA highly induced the expression of *SmCSN5*. SmCSN5, on interaction with SmMYB36, was translocated from the cytoplasm to the nucleus, where its ubiquitination-based degradation was inhibited. Upon exposure to MeJA, the elevated expression of *SmCSN5* promoted the accumulation of SmMYB36, which was biased toward tanshinone production. In summary, the proposed SmCSN5-SmMYB36-target gene module initially resolved the regulatory mechanisms behind tanshinone and phenolic acid biosynthesis at the transcriptional and translational levels and provided a reference for the use of genetic engineering to preferentially enhance the accumulation of compounds with specific bioactivities in *S. miltiorrhiza*.

## Materials and methods

### Plant materials and genetic transformation

Sterilized seedlings of tobacco (*Nicotiana benthamiana*), Arabidopsis (Col-0), *S. miltiorrhiza*, and rice (*Oryza sativa* subsp. *japonica* Nipponbare) were grown in a plant tissue culture (PTC) room under a 16-h light/8-h dark period at 26°C, except during the production of hairy roots, which was conducted under constant darkness. *Salvia miltiorrhiza* wild-type (WT) and transgenic plants with hairy roots were obtained for synchronization using a reported method [72]. Hairy roots were induced in the WT plants by *Agrobacterium tumefaciens* ATCC15834, 0.3 g was weighed and cultured in conical flasks containing 50 ml of 6,7-V base salt with vitamins liquid medium, at 25°C, 120 rpm, in the dark for 3 weeks. For treatments with hormones and replicating the stress-inducing conditions, the hairy roots were transferred to 6,7-V liquid media containing 100 μM MeJA or lacking it (control). Set up three biological replicates by using each vial of hairy roots as one biological replicate (line). The RNA was extracted from the different tissues, such as the roots, stems, leaves, and flowers of five plants grown for a year. Samples were collected after 0, 0.5, 1, 6, 24, 48, and 72 h of treatment and stored at −80°C until RNA extraction. Alternatively, control and *SmCSN5*-RNAi transgenic hairy root lines were cultured for 3 weeks in the same way as described above, and samples were collected for determination of tanshinones and phenolic acids by adding a final concentration of 100 uM MeJA and continuing the culture for 1 week.

### Plasmid construction

To construct the hairy root transformation vector, *SmCSN5* (XP_057766823.1) coding sequence (CDS) full-length sequence 783 bp and designed RNAi sequence 248 bp were cloned, amplified by polymerase chain reaction (PCR), and constructed on the entry vector pDNOR207 using BP clonase. After that, the target genes of the entry vectors were constructed on pK7WG2R and pK7WIWG2R using LR clonase to generate pK7WG2R-*SmCSN5* and pK7WIWG2R-RNAi-*SmCSN5*. These plasmids were constructed using the gateway technology, and the instructions of the BP and LR clonase kits were followed (Invitrogen, Carlsbad, CA, USA). Besides, *SmCSN5* was inserted in the reverse orientation into the pK7WG2R-*SmMYB36* vector, replacing the *NPTII* between the *Nos* promoter and terminator to obtain the double-gene transformation plasmid, pK7WG2R-Anti*SmCSN5*-*SmMYB36*.

For overexpression in Arabidopsis, the fusion-expression vector pCsGFPBT-*SmCSN5*-*GFP* was generated by inserting the complete open reading frame (ORF) of *SmCSN5* into the pCsGFPBT vector. For constructing the vectors used in the yeast two-hybrid (Y2H) experiments, the *SmCSN5*/*SmCSN5*^1–224^/*SmCSN5*^1–99^/*SmCSN5*^100–224^/*SmCSN5*^159–239^ sequences were cloned into the prey vector pGADT7, and the *SmMYB36*^1–153^/*SmMYB36*^1–111^/*SmMYB36*^112–153^ sequences were cloned in the bait vector pGBKT7. For the pull-down experiments, the complete CDSs of *SmMYB36* and *SmCSN5* were inserted into the pGEX4T-1 and pET32a vectors to obtain pGEX4T-1-*SmMYB36/SmCSN5*-*GST* and pET32a-His-*SmCSN5*. For the coimmunoprecipitation (CoIP) experiments, the complete CDSs of SmMYB36 and SmCSN5 were inserted into the N-terminus of the tagged proteins GFP (green fluorescent protein) and Myc, respectively, to generate pCsGFPBT-*SmMYB36-GFP* and pCAMBIA1300-*SmCSN5-*Myc. For colocalization experiments in tobacco leaves, the CDS of SmCSN5 was inserted into the N-terminus of the tagged protein mCherry to obtain pCAMBIA1301-*SmCSN5-mCherry*. For colocalization experiments in rice protoplasts, *SmCSN5* was expressed in fusion with the C-terminal end of the tagged protein mCherry, and *SmMYB36* was expressed in fusion with the C-terminal end of the tagged protein GFP to obtain pBS*-mCherry*-*SmCSN5* and pTF486-*GFP-SmMYB36*. These vectors were constructed using a homologous recombinant ligase (Vazyme Biotech Co. Ltd., Nanjing, China) or T4 DNA ligase (Thermo Fisher Scientific, Waltham, MA, USA). For the luciferase complementation (LCI) and bimolecular fluorescence complementation (BiFC) experiments, *SmMYB36* was fused at the N-terminal end of GFP (nGFP) and LUC (nLUC), and SmCSN5 at the C-terminal end of GFP (cGFP) and LUC (cLUC), respectively, to obtain the vector combinations of cLUC-SmCSN5 + nLUC-SmMYB36 and cGFP-SmMYB36 + cGFP-SmCSN5. The full-length ORF of *SmMYB36* was inserted into pGreen II 62-SK to act as an effector vector, and the pGreenII 0800-*P_SmGAPC_/P_SmRAS_*/*P_SmDXS2_*/*P_SmGGPPS1_*/*P_SmCPS1_*/*P_SmERF6_*/ *P_SmERF115_*-*LUC* was used as a reporter vector in the dual-LUC assay. The vector used for the overexpression of *SmMYB36* in Arabidopsis, pGWB18-Myc-*SmMYB36*, and the prokaryotic expression vector, pET32a-His-*SmMYB36*, used in the cell-free experiments were identical with those published previously [32]. All the primers used are summarized in Supplementary Table S1.

### Identification of SmCSN proteins, multiple sequence comparison, and phylogenetic analysis

First, using CSN1–8 from Arabidopsis as the query sequence, the whole-genome protein library of *S. miltiorrhiza* was scanned, employing the native BLAST tool of TBtools [73], with the E value set to 1e^−10^. The results with ≤50% identity were counted to determine the sequences most similar to the CSN1–8 family of Arabidopsis. Second, the unique sequences were uploaded to the Pfam database, while the SMART database was accessed to ascertain whether the SmCSNs possessed the corresponding conserved structural domains.

Using the software MEGA7, subunits 1–8 of the COP9 signaling complex Arabidopsis were selected for phylogenetic analysis with *SmCSNs*/*SmCSN5* using the 1000-bootstrap replication, neighbor-joining (NJ) method. DNAMAN was chosen to align the amino acid sequences of AtCSN5A and AtCSN5B, which were shown to be in the same clade as SmCSN5 by phylogenetic analysis.

### Yeast two-hybrid assays

The plasmid combinations of pGADT7-*SmMYB36*^1–153^ + pGBKT7-*SmCSN5*, pGADT7 + pGBKT7-*SmCSN5*, and pGADT7-*SmMYB36*^1–153^ + pGBKT7 were cotransferred into yeast Y2Hgold recipient cells. PCR identified positive colonies after 2–3 days of incubation on the double-deficient medium SD/−Trp-Leu. Positive transformants were cultured in SD/−Trp-Leu liquid medium until the cell density reached an OD_600_ = 0.6, and the growth was observed for 2–3 days by pipetting the liquid medium onto the quadruple-deficient medium SD/−Leu-Trp-His-Ade/AbA (125 ng/ml)/X-α-Gal (4 mg/ml).

### LCI and BiFC assays

The plasmids constructed for the LCI experiments, cLUC-*SmCSN5* and nLUC-*SmMYB36*, and the empty plasmid were used to transform the *A. tumefaciens* GV3101 (pSoup-p19) cells separately. The tobacco leaves were injected with different combinations of the bacterial solutions and incubated for 3–5 days. The tobacco leaves were then cut and laid flat on 4% agar plates, protected from light, evenly coated with luciferase substrate, and placed in a dark environment for 5 min. Then, they were imaged using the Lumazone Pylon 2048B live plant molecular marker imaging system (Princeton, NJ, USA) to obtain the dark-field and bright-field images separately.

The plasmids constructed for the BiFC assay were divided into an experimental group (nGFP-SmMYB36 and cGFP-SmCSN5) and a control group (nGFP and cGFP) for the polyethylene glycol (PEG)-mediated transformation of Arabidopsis protoplasts, respectively. Arabidopsis protoplasts were isolated and transformed based on the previously described methods [74]. Lastly, the protoplasts were aspirated and placed under a TCS SP8 laser scanning confocal microscope (LSCM) (Leica, Wetzlar, Germany). The fluorescence signal was observed at an emission wavelength of 507 nm and an excitation wavelength of 488 nm.

### Protein purification and *in vitro* pull-down assay

The fusion-expression plasmids His-*SmMYB36*, His*-SmCSN5*, and *GST*-*SmMYB36* were employed to transform *Escherichia coli* E25566 to induce gene expression and purify the expressed protein. The conditions for the induction of expression were 0.3 mM IPTG at 16°C and 16 h for the His-SmCSN5 recombinant protein and 0.8 mM IPTG at 16°C and 24 h for the GST-SmMYB36 and His-SmMYB36 fusion proteins. The broth containing the completely induced bacteria was collected, and the cells were crushed using a JN-02C low-temperature, ultra-high pressure continuous flow cell disrupter (Guangzhou Juneng Biology & Technology Co. Ltd., Guangzhou, China), and fusion proteins were purified using His60 Ni Superflow Resin and GST 4FF prepacked gravity column (Sangon Biotech, Shanghai, China).

The His-SmCSN5 and GST-SmMYB36 fusion proteins were mixed and incubated at 4°C for 4–6 h. The mixture was then moved to a GST purification column and incubated overnight at 4°C. Finally, the eluate was added to 1× SDS loading buffer, boiled for l0 min, and western blotting was carried out with the anti-His and anti-GST antibodies (Sangon Biotech, Shanghai, China).

### CoIP and *in vivo* ubiquitination assays

The CoIP experiments were performed in the tobacco transient transformation system, GV3101 (pSoup-p19), containing the *SmCSN5*-Myc, *SmMYB36*-*GFP*, and empty plasmids were mixed into the experimental (*SmCSN5*-Myc + *SmMYB36*-*GFP*) and control (*SmCSN5*-Myc + *GFP*) groups and injected into tobacco leaves. Nondenaturing and denaturing lysis buffers from the universal Pierce™ Classic IP Kit (Thermo Fisher Scientific, Waltham, MA, USA) were used to extract the proteins for the IP assay and western blotting samples, respectively. IP of SmCSN5-Myc was performed using Pierce™ protein A/G plus agarose coincubated with an anti-Myc antibody (Abways, Shanghai, China), and western blotting analysis was performed using labeled antibodies (anti-GFP and anti-Myc) and anti-UBQ (Sigma-Aldrich, St Louis, MO, USA) [43].

### Subcellular colocalization of SmCSN5 and SmMYB36

Solutions containing the transformed GV3101 (pSoup-p19) bacterial cells carrying the fluorescent tag SmCSN5-mCherry and SmMYB36-GFP plasmids, and the empty plasmids were injected into the leaves of tobacco seedlings in different combinations, and cultured for ~3 days. Rice protoplasts were transformed into rice protoplasts by transforming GV3101 (pSoup-p19) bacterial cell solution containing the plasmid carrying the fluorescent tag mCherry-SmCSN5 + GFP-SmMYB36, and the empty plasmid combination. Protoplasts were isolated from 7- to 14-day-old rice seedlings and transformed according to the method in the Rice Protoplast Preparation and Transformation Kit (Coolaber, Beijing, China). Then, the green and red fluorescence were photographed at excitation wavelengths of 488 and 580 nm and emission wavelengths of 507 and 610 nm, respectively, using a TCS SP8 bio-laser confocal microscope (Leica, Wetzlar, Germany).

### Cell-free degradation assay

For the *in vitro* SmMYB36^1–153^-ubiquitination-based degradation experiments, the purified His-SmMYB36^1–153^ protein was added to a degradation reaction solution containing 0.1% DMSO, and the changes in the content of the SmMYB36^1–153^ protein in relation with reaction time were analyzed at the time points of 0, 5, 15, 30, 60, and 90 min. The composition of the degradation buffer and the method of extraction of total proteins from *S. miltiorrhiza* are based on those mentioned in a previous study [75]. The same procedure was used for the ubiquitinated-based degradation experiments of SmMYB36 in the transgenic Arabidopsis and tobacco plants transiently overexpressing *SmCSN5*, WT plants, and those transformed with an empty vector, which were used as controls.

### *In vivo* assay of protein degradation

Cells of the transient expression system, GV3101 (Soup-p19), containing the *SmMYB36*-*GFP* were used to infect tobacco leaves for two and a half days and then treated with 0.1% DMSO (control), 100 μM cycloheximide (CHX), 50 μM MG132, and 100 μM CHX + 50 μM MG132, separately. The total proteins of these tobacco leaves were extracted 3 days later and detected using anti-SmMYB36 and anti-RuBisCo antibodies through western blotting, with RuBisCo as the reference. Transgenic Arabidopsis seedlings overexpressing Myc-*SmMYB36* were obtained and cultured under standard conditions on a half-MS medium for 14 days. They were then subjected to the four treatments: 0.1% DMSO, 100 μM CHX, 50 μM MG132, and 100 μM CHX + 50 μM MG132 for 6 h. Total proteins were extracted, and western blotting was performed employing the anti-SmMYB36 and antiactin antibodies, with actin as the reference protein.

### Dual-luciferase assay

The reporter plasmids *P_SmGAPC_*::*LUC*, *P_SmRAS_*::*LUC*, *P_SmDXS2_*::*LUC*, *P_SmGGPPS1_*::*LUC*, *P_SmCPS1_*::*LUC*, *P_SmERF6_*::*LUC*, and *P_SmERF115_*::*LUC*; and the effector plasmids *SmMYB36* and *SmCSN5*-*GFP* were mixed in different combinations and injected into tobacco leaves. The luminescence was determined based on a previous method [32], and the method used to detect luminescence in the live transformed leaves was consistent with the technique employed for LCI.

### Estimation of phenolic acids and tanshinones by HPLC (high performance liquid chromatography)

All the transgenic hairy-rooted and WT plants were collected to extract tanshinones and phenolic acids. The samples were extracted, and the HPLC-based measurements were performed individually with three biological and technical replicates, exactly as mentioned in a previous report [72, 76]. The chromatographic conditions were as follows: binary gradient elution, flow rate of 1 ml/min, column temperature of 35°C, injection volume of 10 μl, and detection wavelengths of 270 and 288 nm for tanshinone and phenolic acid, respectively. Total phenolic acid (TPA) was considered equal to the sum of Sal B (Salvianolic acid B) and RA, while total tanshinone (TTA) to that of cryptotanshinone (CT), tanshinone IIA (TIIA), tanshinone I (TAI), and dihydrotanshinone I (DTI).

### qRT-PCR (quantitative reverse transcription PCR)

Total RNA was extracted from the transgenic hairy roots and WT plants using the total plant RNA extraction kit (Tiangen, Beijing, China) as per the instructions provided. The integrity and concentration of the RNA were ascertained using 1% agarose gel electrophoresis and a Nano Drop™ ultramicroscopic spectrophotometer (Thermo Fisher Scientific, Waltham, MA, USA). Then, the EvoM-MLV Reverse Transcription Kit (Accurate Biotechnology, Hunan, China) was used to reverse transcribe total RNA to cDNA. qRT-PCR was performed using a SYBR Premix Ex Taq kit (TaKaRa, Dalian, China). We used *SmActin* and *SmUbiquitin* as reference genes for normalization, setting up three biological replicates and three technical replicates. *SmActin* and *SmUbiquitin* were identified as the most stably expressed in various tissues of *S. miltiorrhiza* [77], and were consequently used as reference genes for normalization. The experiment was set up with three biological replicates and three technical replicates. The primers used are shown in Supplementary Table S2. *SmUbiquitin* was used as the housekeeping gene, and the relative expression of each gene is listed in Supplementary Fig. S1.

## Supplementary Material

Web_Material_uhaf005

## Data Availability

Detailed data supporting the findings of this study can be found in the supplementary material.
