# Supplementary material for: SmCSN5 is a synergist in the transcription factor SmMYB36-mediated biosynthesis of tanshinones and phenolic acids in Salvia miltiorrhiza
Source: Hortic Res. 2025 Jan 6;12(4):uhaf005. doi: 10.1093/hr/uhaf005 (PMC11896976; doi:10.1093/hr/uhaf005)
Supplement: Web_Material_uhaf005 [file web_material_uhaf005.zip › Figure S1-S7 + Table S1-S2.pdf]

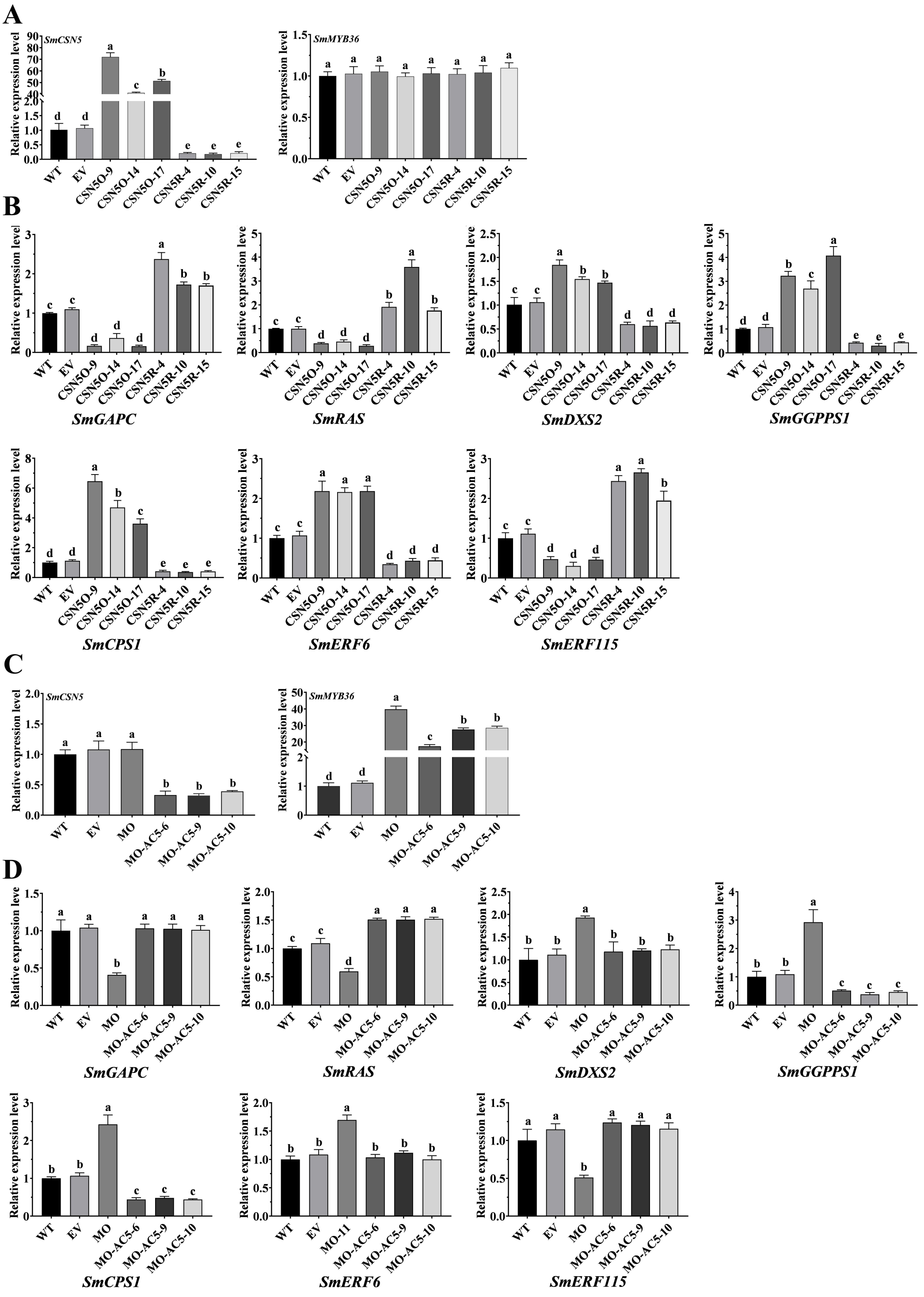

Figure S1. Relative expression of each gene using *SmUbiquitin* as a reference control. The different letters: a, b, c, d, and e represent the statistical significance of the differences in the results calculated using one-way ANOVA and Duncan's test at $P < 0.05$.

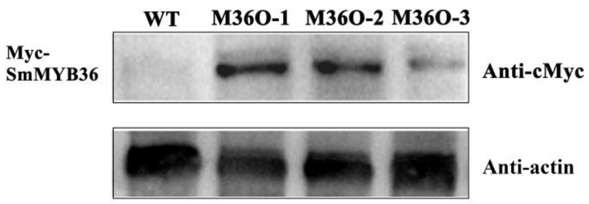

Figure S2. Total proteins of *SmMYB36*-transgenic Arabidopsis plants were extracted and immunoblotted using anti-Myc antibodies, respectively; WT was used as a negative control.

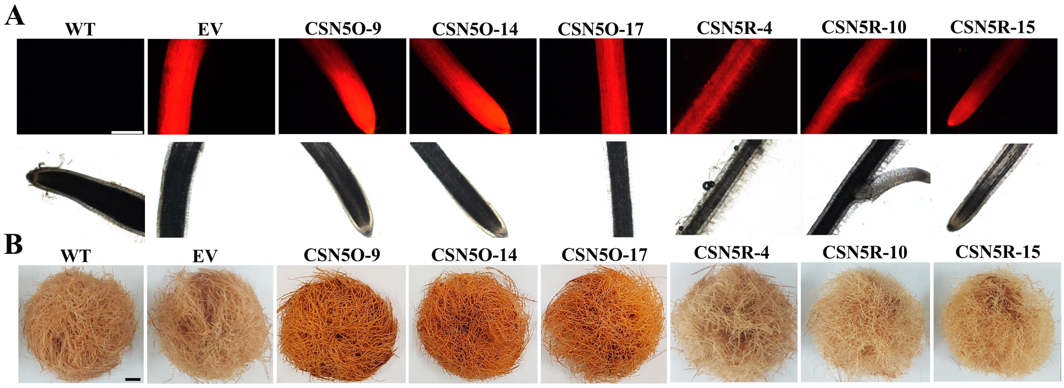

Figure S3. **(A)** Red fluorescent protein assays were performed on the *SmCSN5*-overexpression and -RNAi hairy root lines using EV and WT as the positive and negative controls, respectively. **(B)** Phenotypes of *SmCSN5*-overexpression and -RNAi hairy root lines. Scale bars represent 500 μm and 5 mm, respectively.

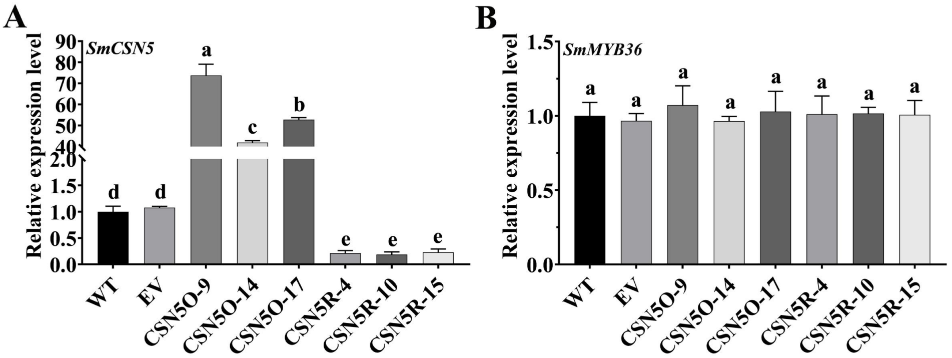

Figure S4. Expression analysis of *SmCSN5* and *SmMYB36* in *SmCSN5*-overexpressing/RNAi plants and control (EV and WT) hairy root lines. Error bars represent SD (n = 3). *SmActin* was used as an internal reference. The different letters: a, b, c, d, and e represent the statistical significance of the differences in the results calculated using one-way ANOVA and Duncan's test at $P < 0.05$.

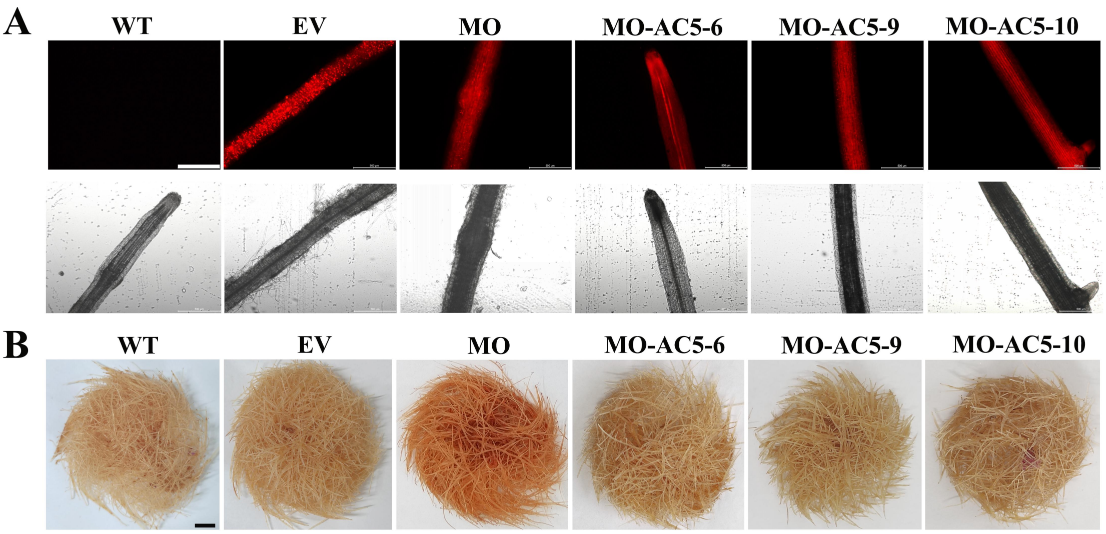

Figure S5. (**A**) Red fluorescent protein detection in *SmMYB36*-overexpression (MO) and double gene coexpression (MO-CA5) hairy root lines. WT and EV were used as the negative and positive controls, respectively. (**B**) Phenotypes of MO and MO-CA5 hairy root lines. Scale bars represent 500 µm and 5 mm, respectively.

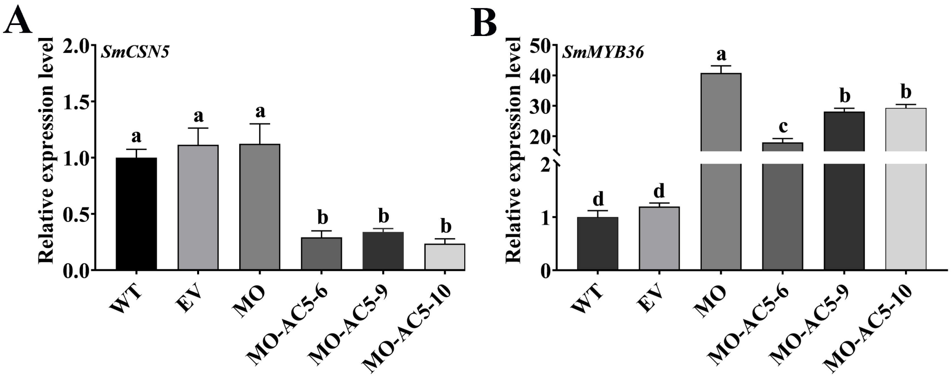

Figure S6. (**A-B**) Expression levels of *SmMYB36* and *SmCSN5* were ascertained in the *SmMYB36*-overexpression (MO), *SmMYB36*-overexpression with concurrent *SmCSN5*-antisense expression (MO-AC), and control (EV and WT) hairy root lines. Error bars represent SD (n=3). *SmActin* was employed as an internal reference. The different letters: a, b, c, and d represent the statistical significance of the differences in the results calculated using one-way ANOVA and Duncan's test at $P < 0.05$.

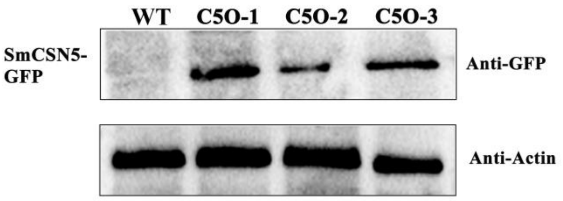

Figure S7. Total proteins of the *SmCSN5*-transgenic Arabidopsis plants were extracted and

immunoblotted using the anti-GFP antibodies, respectively. WT was used as a negative control.

Table S1 Primer sequences for vector construction

Primer name	Sequence (5' to 3')	Function
BD-SmCSN5-F	GCCATGGAGGCCGAATTCATGGACGCTTTTGCCCTC	Clonin pGBKT7-SmCSN5
BD-SmCSN5-R	CGGCCGCTGCAGGTCGACTCAGGATTCAATCATAGGTTCAGG	Clonin pGBKT7-SmCSN5
BD-SmMYB36$^{1-111}$-F	GCATATGGCCATGGAGGCCGAATTCATGGCGAGTGATGCATCTCTGAAC	Cloning pGBKT7-SmMYB36$^{1-111}$
BD-SmMYB36$^{1-111}$-R	GCGGCCGCTGCAGGTCGACGGATCCTCAGGCTTTCTTCCTCAAGTGAGCATT	Cloning pGBKT7-SmMYB36$^{1-111}$
BD-SmMYB36$^{112-153}$-F	GCATATGGCCATGGAGGCCGAATTCATGTTGATGGACAAGTTACCCGC	Cloning pGBKT7-SmMYB36$^{112-153}$
BD-SmMYB36$^{112-153}$-R	GCGGCCGCTGCAGGTCGACGGATCCTCAAAAACTTTTAACCCAATCCAACCCAGAG	Cloning pGBKT7-SmMYB36$^{112-153}$
AD-SmMYB36$^{1-153}$-F	TATGGCCATGGAGGCCAGTGAATTCATGGCGAGTGATGCATCTCTGA	Cloning pGADT7-SmMYB36$^{1-153}$
AD-SmMYB36$^{1-153}$-R	ATCTACGATTCATCTGCAGCTCGAGCTCAAAAACTTTTAACCCAATCCAACCCAGAGG	Cloning pGADT7-SmMYB36$^{1-153}$
AD-SmCSN5$^{1-99/224}$-F	TATGGCCATGGAGGCCAGTGAATTCATGGACGCTTTTGCCCTCC	Cloning of pGADT7-SmCSN5$^{1-99}$ and pGADT7-SmCSN5$^{1-224}$, respectively
AD-SmCSN5$^{1-99}$-R	ATCTACGATTCATCTGCAGCTCGAGTCATTCTGGATATGTCCTGAAAGCAC	Clonin pGADT7-SmCSN5$^{1-99}$
AD-SmCSN5$^{1/100-224}$-R	ATCTACGATTCATCTGCAGCTCGAGTCACTGCTCTACAGTTATCTTGGCAC	Cloning of pGADT7-SmCSN5$^{1-224}$ and pGADT7-SmCSN5$^{100-224}$, respectively
AD-SmCSN5$^{159-239}$-F	TATGGCCATGGAGGCCAGTGAATTCTCTTCTTCTCCTTTGCTTGGTAACGG	Cloning pGADT7-SmCSN5$^{159-239}$
AD-SmCSN5$^{159-239}$-R	ATCTACGATTCATCTGCAGCTCGAGTCAATTAAAAAGGATATCCTTGATAACCTGTG	Cloning pGADT7-SmCSN5$^{159-239}$
AD-SmCSN5$^{100-224}$-F	TATGGCCATGGAGGCCAGTGAATTCGGCTACAAACCCCCAGATGAAC	Cloning pGADT7-SmCSN5$^{100-224}$
LUCc-SmCSN5-F	GGGGACGAGCTCGGTACCATGGACGCTTTTGCCCTCC	Cloning cLUC-SmCSN5
LUCc- SmCSN5-R	GTACGAGATCTGGTCGACGGATTCAATCATAGGTTCAGGACCG	Cloning cLUC-SmCSN5
LUCn-SmMYB36-F	GGGGACGAGCTCGGTACCATGGCGAGTGATGCATCTCTGA	Cloning nLUC-SmMYB36

LUCn- SmMYB36-R	GTACGAGATCTGGTCGACTTCATCCTCGTCGAGTT CAAG	Cloning nLUC-SmMYB36
GFPn-SmMYB36-F	GACGAGCTGTACAAGCATATGATGGACGCTTTTGC CCTCC	Cloning 35S::nGFP-SmMYB36
GFPn-SmMYB36-R	CGCCACAACATCGAGGACCATATGATGGCGAGTG ATGCATCTCTGA	Cloning 35S::nGFP-SmMYB36
GFPc-SmCSN5-F	GACGAGCTGTACAAGCATATGATGGACGCTTTTG CCCTCC	Cloning 35S:cGFP-SmCSN5
GFPc-SmCSN5-R	CAACAGGATTCAATCTTAAGTCAGGATTCAATCA TAGGTTCAGGACC	Cloning 35S:cGFP-SmCSN5
Pet32a-SmMYB36$^{1-153}$-F	GACAGCAAATGGGTCGCGGATCCATGGCGAGTGA TGCATCTCTGA	Cloning pET32a-His-SmMYB36$_{1-153}$
Pet32a-SmMYB36$^{1-153}$-R	GAGTGCGGCCGCAAGCTTGTCGACAAAACTTTTA ACCCAATCCAACCCAGAGG	Cloning pET32a-His-SmMYB36$_{1-153}$
pGEX6P-1-SmCSN5-F	TCCAGGGGCCCCTGGGATCCATGGACGCTTTTGCC CTC	Cloning pGEX6P-1-GST-SmCSN5
pGEX6P-1-SmCSN5-R	CGAGTCGACCCGGGAATTCTCAGGATTCAATCAT AGGTTCAGG	Cloning pGEX6P-1-GST-SmCSN5
pGEX6P-1-SmMYB36-F	TCCAGGGGCCCCTGGGATCCATGGCGAGTGATGC ATCTCTG	Cloning pGEX6P-1-GST-SmMYB36
pGEX6P-1-SmMYB36-R	CGAGTCGACCCGGGAATTCTCATTCATCCTCGTCG AGTTCAAGA	Cloning pGEX6P-1-GST-SmMYB36
pCAMBIA1301-SmCSN5-F	CGTCTAGAATGGCGAGTGATGCATCTCTGA	Cloning pCAMBIA1301-SmCSN5-mCherry
pCAMBIA1301-SmCSN5-R	CGGGTACCTCATTCATCCTCGTCGAGTTCAAG	Cloning pCAMBIA1301-SmCSN5-mCherry
pK7WG2R-PDNOR207-SmCSN5-F	GGGGACAAGTTTGTACAAAAAAGCAGGCTTAATG GACGCTTTTGCCCTCCC	Cloning PK7WG2R-SmCSN5
pK7WG2R-PDNOR207-SmCSN5-R	GGGGACCACTTTGTACAAGAAAGCTGGGTACTAA GGTAATAACTCTGAAACTTGA	Cloning PK7WG2R-SmCSN5
pK7GWIWG2R-RNAi-CSN5-F	GGGGACAAGTTTGTACAAAAAAGCAGGCTTACAA AATAGACTGGATCTGGGACTTA	Cloning pK7WIWG2R-RNAi-SmCSN5
pK7GWIWG2R-RNAi-CSN5-R	GGGGACCACTTTGTACAAGAAAGCTGGGTAAGCC TCCATTACCAACACCTCA	Cloning pK7WIWG2R-RNAi-SmCSN5
pCsGFPBT-SmMYB36-F	TACGAACGATAGCCATGGCGAGTGATGCATCTCTG	Cloning pCsGFPBT-SmMYB36-GFP
pCsGFPBT-SmMYB36-R	CTCCTCGCCCTTGCTCACTTCATCCTCGTCGAGTT CAAGA	Cloning pCsGFPBT-SmMYB36-GFP
pCAMBIA1300-SmCSN5-F	GAAGAGGACTTGAATTCGGTACCCATGGACGCTT TTGCCCTC	Cloning pCAMBIA1300-SmCSN5-Myc
pCAMBIA1300-SmCSN5-R	GCGTCCTAGGCTACGTAGGATCCTCAGGATTCAA TCATAGGTTCAGG	Cloning pCAMBIA1300-SmCSN5-Myc

pCsGFPBT-SmCSN5-F	TACGAACGATAGCCATGATTGATTACCGCACGCC	Cloning pCsGFPBT-SmCSN5-GFP
pCsGFPBT-SmCSN5-R	CTCCTCGCCCTTGCTCACTCTAATCTCAGCAACTT TAGA	Cloning pCsGFPBT-SmCSN5-GFP
pTF486-SmMYB36-F	GCTGTACAAGTAAAGCGGCCGCATGGCGAGTGAT GCATCTCTG	pTF486-GFP-SmMYB36
pTF486-SmMYB36-R	GATCTGCAGCCGGGCGGCCGCTTCATCCTCGTCG AGTTCAAGA	pTF486-GFP-SmMYB36
pBS-SmCSN5-F	CTGTACAAGTAAAGCGGCCGCATGGACGCTTTTG CCCTC	pBS-mCherry-SmCSN5
pBS-SmCSN5-R	AACGATCGCCGGGCGGCCGCTTCAATCATAGGTT CAGGAC	pBS-mCherry-SmCSN5

Table S2 Primer sequences for vector construction

Primer name	Sequence (5' to 3')
qSmMYB36-F	GGCTCAACTATCTTAGTCCTGAT
qSmMYB36-R	CCTCCGGTTTCATTCCAAATGT
qSmCSN5-F	GGCCAGAGAATGTGGTAGGATGG
qSmCSN5-R	AGCAGAAACAGTCCTTGTCGGA
qSmDXS2-F	GCCCGTGGGAGCCCTCAG
qSmDXS2-R	AGTCATGCCCTTTGCTGCTTCG
qSmGGPPS1-F	ACAAGACCACGTATCCCAAGC
qSmGGPPS1-R	TCTGCCTATGTGCAATGTAATCG
qSmERF6-F	TCATCCGACCCGAACCGACCCGATT
qSmERF6-R	AACGCCGCCACGCCATCACAGGAT
qSmERF115-F	TTACGCCAACAACCACCACCAC
qSmERF115-R	CCCACCGCCGTAGTGAAAATCC
qSmCPS1-F	CCACATCGCCTTCAGGGAAGAAAT
qSmCPS1-R	TTTATGCTCGATTTCGCTGCGATCT
qSmRAS-F	CATCCGATCCACTTTTTCATAGACG
qSmRAS-R	AATTAGTGTTGTAGGAGGCAGGAG
qActin2-F	CTGGAATGGTGAAGGCTGGTT
qActin2-R	CGATTGGATACTTCAGAGTGAGGAT
qSmGAPC-F	GAGCTCGTTGCTGTGAATGA
qSmGAPC-R	CAAACTCAGCACCAGCTTCA

qSmUbiquitin-F	GTTGATTTTTGCTGGGAAGC
qSmUbiquitin-R	GATCTTGGCCTTCACGTTGT
