# Supplementary figures and images for: SmCSN5 is a synergist in the transcription factor SmMYB36-mediated biosynthesis of tanshinones and phenolic acids in Salvia miltiorrhiza
Source: Hortic Res. 2025 Jan 6;12(4):uhaf005. doi: 10.1093/hr/uhaf005 (PMC11896976; doi:10.1093/hr/uhaf005)

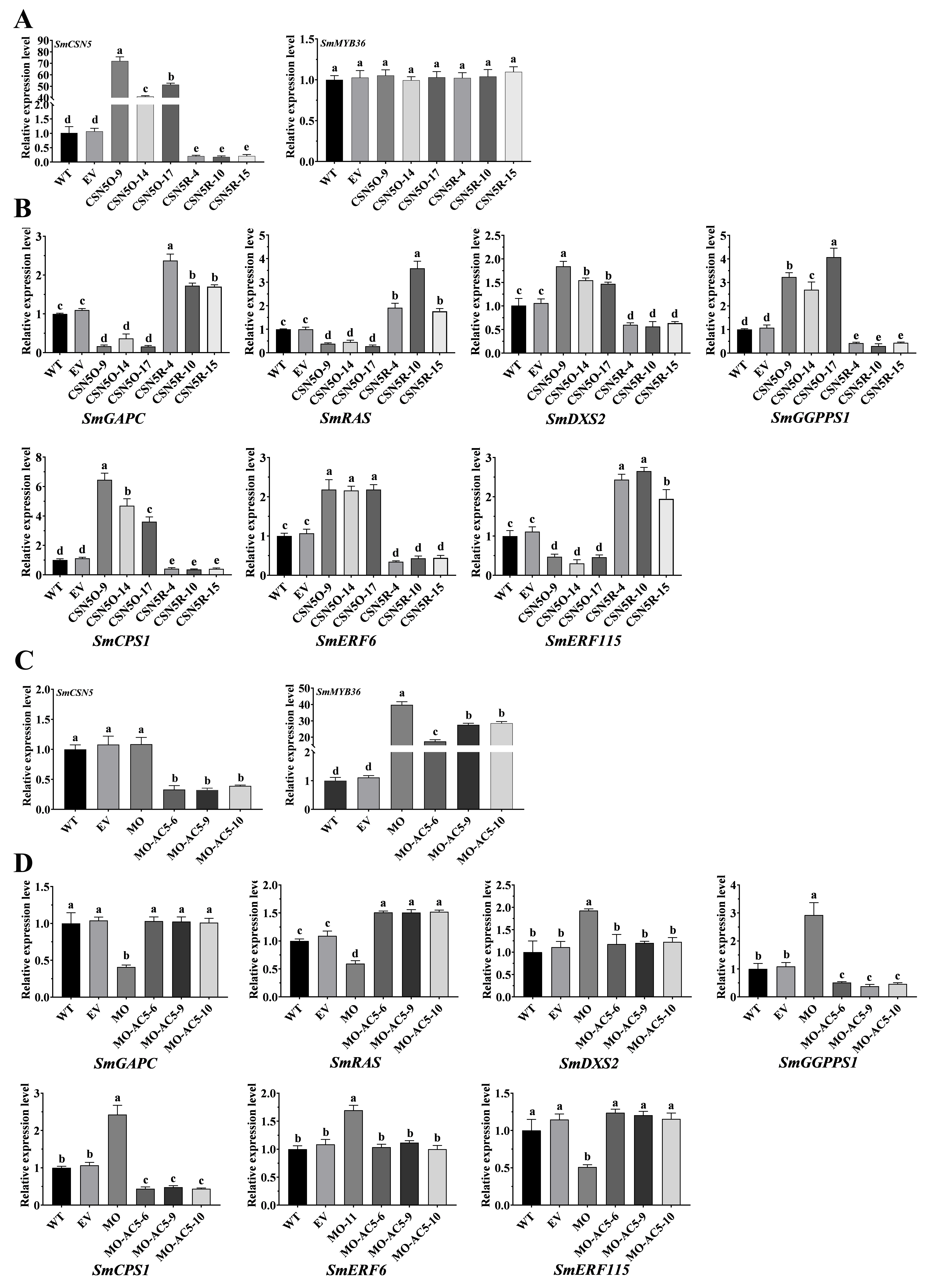

Supplement: Web_Material_uhaf005 [file web_material_uhaf005.zip › Figure S1.tif]

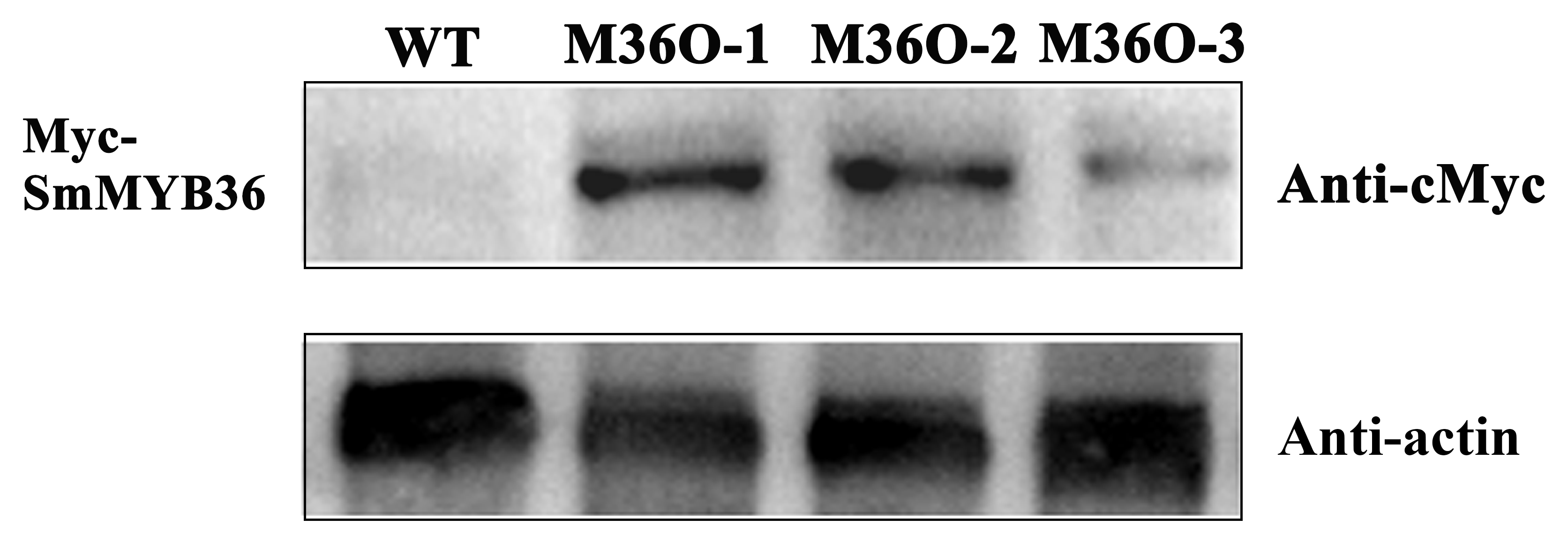

Supplement: Web_Material_uhaf005 [file web_material_uhaf005.zip › Figure S2.tif]

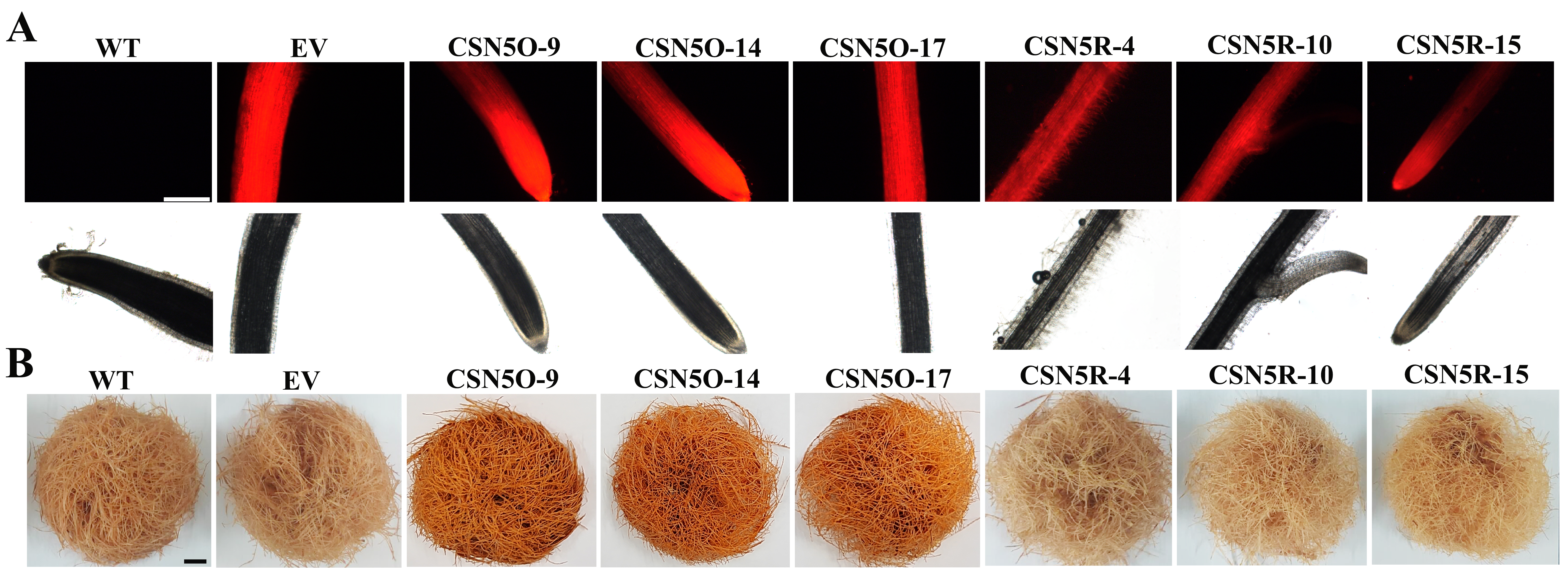

Supplement: Web_Material_uhaf005 [file web_material_uhaf005.zip › Figure S3.tif]

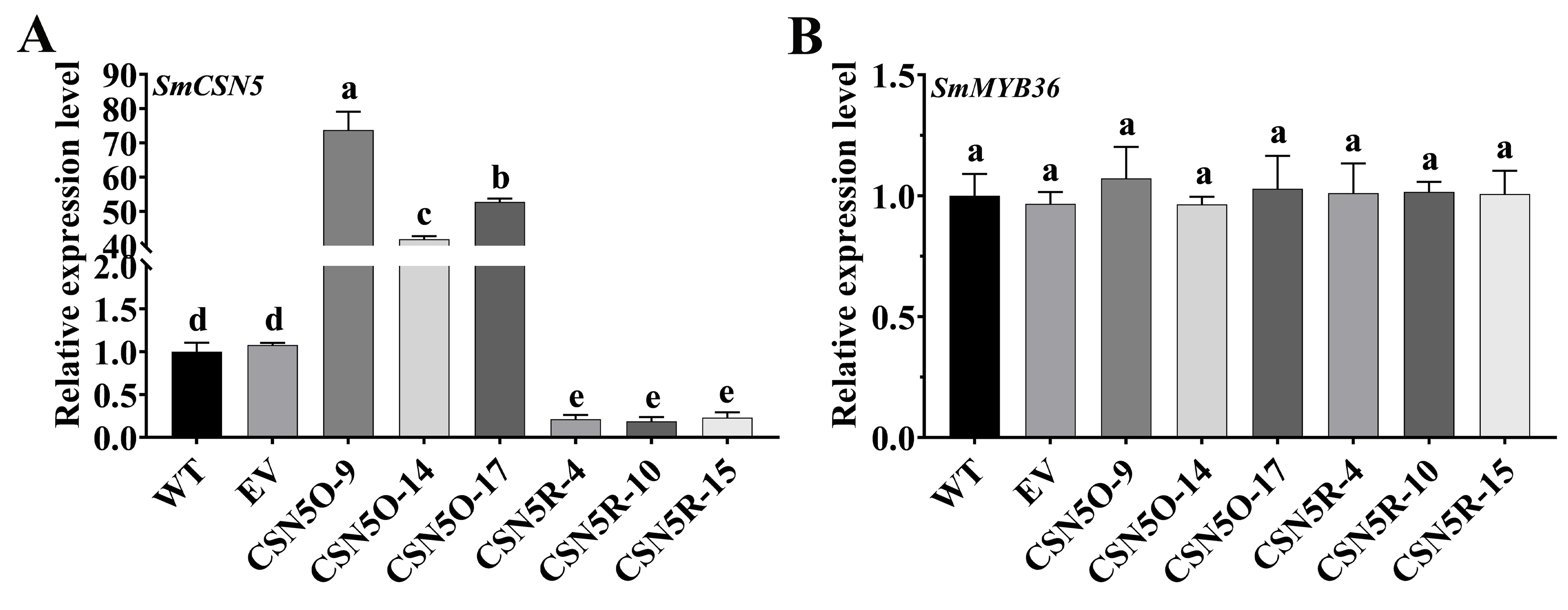

Supplement: Web_Material_uhaf005 [file web_material_uhaf005.zip › Figure S4.tif]

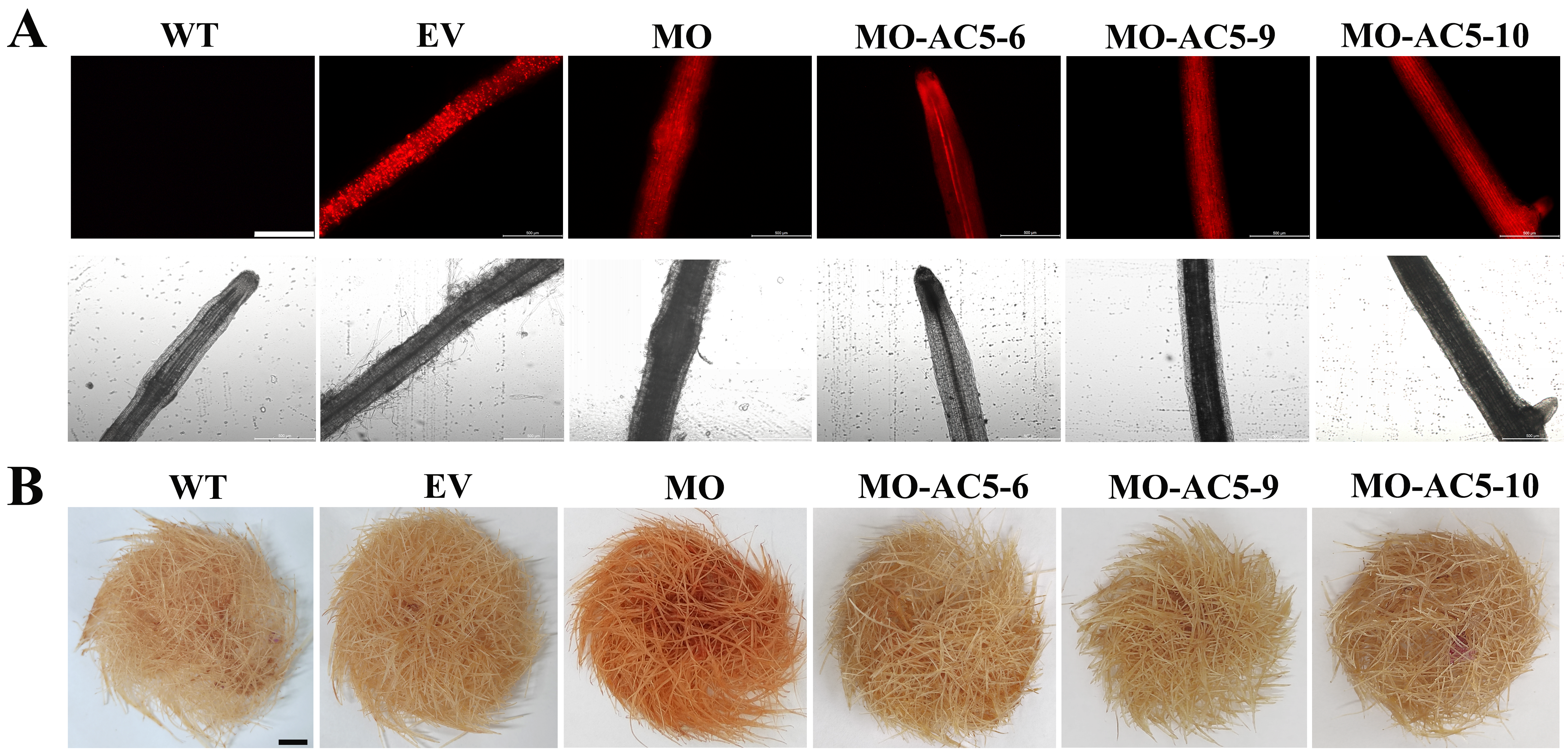

Supplement: Web_Material_uhaf005 [file web_material_uhaf005.zip › Figure S5.tif]

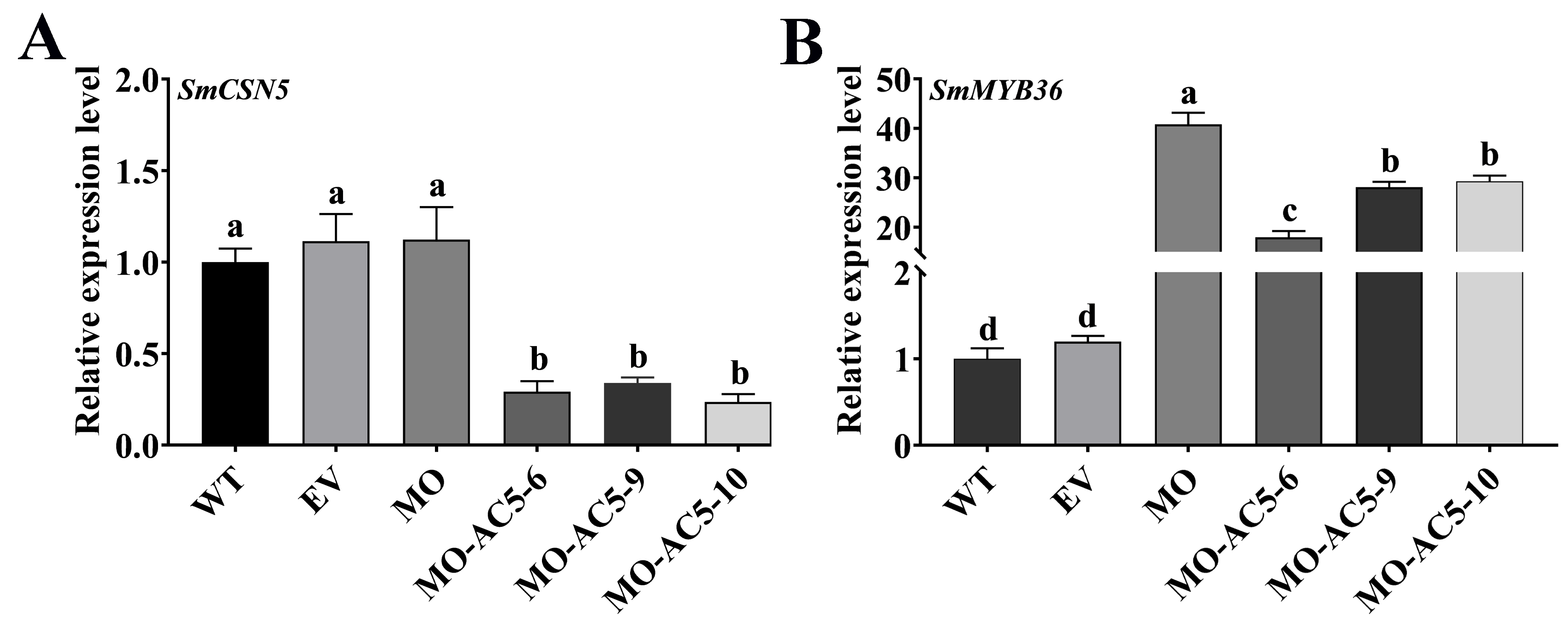

Supplement: Web_Material_uhaf005 [file web_material_uhaf005.zip › Figure S6.tif]

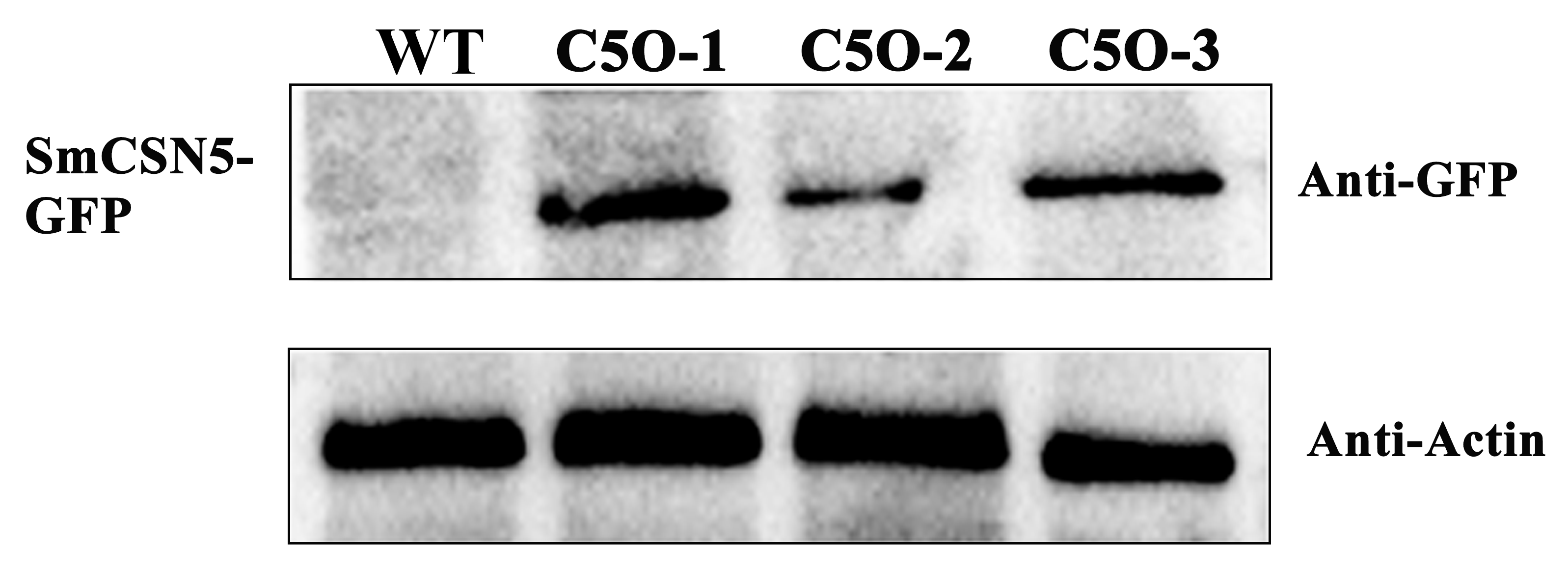

Supplement: Web_Material_uhaf005 [file web_material_uhaf005.zip › Figure S7.tif]
